# Supplementary material for: Examining the latent structure and correlates of sensory reactivity in autism: a multi-site integrative data analysis by the autism sensory research consortium
Source: Mol Autism. 2023 Aug 28;14:31. doi: 10.1186/s13229-023-00563-4 (PMC10464466; doi:10.1186/s13229-023-00563-4)
Supplement: Supplementary file 1 — Additional file 1. Supplemental methods and tables. [file 13229_2023_563_MOESM1_ESM.pdf]

Examining the Latent Structure and Correlates of Sensory Reactivity in Autism:

A Multi-site Integrative Data Analysis by the Autism Sensory Research Consortium

Zachary J. Williams *et al.* (2023)

Supplemental Information

Table of Contents

Supplemental Methods ***S2***
Constructs and Measures **S2**
Data Analysis **S7**
Supplemental Table S1 ***S10***
Supplemental Table S2 ***S12***
Supplemental Table S3 ***S15***
Supplemental Table S4 ***S16***
Supplemental Table S6 ***S18***
Supplemental Table S7 ***S28***
Supplemental References ***S29***

Supplemental Methods

Constructs and Measures

Measures of Sensory Reactivity

(Short) Sensory Profile 1. The Sensory Profile 1 (SP1; Dunn, 1999) is a 125-item caregiver-report questionnaire designed to assess a child's sensory reactivity across multiple sensory modalities. Items are organized into four theoretically driven "sensory quadrants" (Low Registration [i.e., HYPO], Sensation Seeking [i.e., SEEK], Sensory Sensitivity [i.e., an aspect of HYPER in which an individual reports discomfort or aversion], and Sensation Avoiding [i.e., an aspect of HYPER in which an individual tends to display an "active" response and avoid the stimulus]). SP1 items are scored on a 5-point Likert scale ranging from 1=*Always* to 5=*Never*, with lower scores indicating a higher frequency of divergent (i.e., non-normative) sensory behaviors. An abbreviated version of the SP1, the Short Sensory Profile 1 (SSP1; McIntosh et al., 1999) is also available, comprising 38 of the original 125 SP1 items as rated on the same 5-point Likert scale. The SP1 and SSP1 were originally designed for children aged 3–10 years, although they are frequently administered to autistic children outside of that age range (Burns et al., 2017; Williams, 2020). Given its relative brevity and commercial availability, the SSP1 is among the most widely used measures of sensory reactivity in autism research to date (Burns et al., 2017; Williams, 2020), and its psychometric properties have been examined in multiple large samples of autistic children and youth (Dwyer et al., 2022; Parks et al., 2020; Tillmann et al., 2020; Tomchek et al., 2014; Williams et al., 2018). As the SP1/SSP1 were the only measures wherein lower scores indicated more significant divergence from a non-autistic sensory profile, items on the SP1/SSP1 were reverse-coded for analyses in order to be consistent in their scoring with the remainder of the sensory questionnaires.

(Short) Sensory Profile 2. The SP1 and SSP1 were revised by the measures' authors in 2014 to create the Sensory Profile 2 and Short Sensory Profile 2 (SP2 and SSP2; Dunn, 2014), the most recent iterations of these questionnaires. The SP2 and SSP2 are composed of 86 and 34 items, respectively, many of which are carried forward or minimally changed from the SP1, and all of which are rated on a 5-point Likert scale from 1 = *Almost Never* to 5 = *Almost Always*. Items are again organized into the four theoretically motivated sensory quadrants specified above. The recommended age range for the SP2 and SSP2 is 3–14 years (Dunn, 2014). Notably, as the SP2 and SSP2 have not been thoroughly validated in samples of autistic children, these measures are infrequently used in the autism literature (Lane et al., 2022; Schulz & Stevenson, 2019; Simpson et al., 2019), and many autism researchers continue to administer the SP1/SSP1 (Burns et al., 2017; Williams, 2020). In the current study, analogous items on the SP1 and SP2 (and their short forms) were combined into single items for the purpose of cross-dataset analyses.

Sensory Experiences Questionnaire, version 2.1. The Sensory Experiences Questionnaire version 2.1 (SEQ-2.1; Baranek, 1999) is a caregiver-report questionnaire that assesses a child's behavioral responses to a range of everyday sensory stimuli. The SEQ-2.1 includes 36 quantitative items that assess the frequency of a child's specific behaviors on a 5-point response scale ranging from 1 = *Almost Never* to 5 = *Almost Always*. Items are classified as representing one of three sensory response patterns (HYPER, HYPO, or SEEK), one of five sensory modality categories (Auditory, Visual, Tactile, Gustatory/Olfactory, and Vestibular/Proprioceptive), and one of two contexts (social and non-social). The SEQ-2.1 was designed for children 6 months through 6 years of age (Baranek, 1999; Baranek et al., 2006), although it has been administered to autistic individuals across a wider age range (Cascio et al., 2015, 2016; Donkers et al., 2015; Feldman et al., 2020; Foss-Feig et al., 2012). After the SP and

related measures, the SEQ-2.1 is the next most commonly used measure of sensory reactivity reported in the autism literature (Burns et al., 2017). The psychometric properties of the SEQ-2.1 have recently been comprehensively evaluated in a large community sample of young children (Lee et al., 2022), but formal factor-analytic investigations of this version of the SEQ in autism-only samples have not been published. However, some psychometric data have been published on an earlier version of the questionnaire with many overlapping items (SEQ-1.0; Baranek et al., 2006; Little et al., 2011), and existing studies incorporating the SEQ-2.1 in autistic individuals have found evidence of the measure's convergent and concurrent validity in this population (e.g., Boyd et al., 2010; Feldman et al., 2021; Watson et al., 2011).

Sensory Experiences Questionnaire, version 3.0. The Sensory Experiences Questionnaire version 3.0 (SEQ-3.0; Ausderau et al., 2014; Baranek, 2009) is an updated version of the SEQ-2.1 that comprises 105 items, 97 of which are quantitative items rated on a 5-point scale ranging from 1 = *Never/Almost Never* to 5 = *Always/Almost Always* across four response patterns (HYPER, HYPO, SEEK, and enhanced perception [EP]), six modality categories (Auditory, Visual, Tactile, Gustatory/Olfactory, Vestibular/Proprioceptive, and Multisensory), and two contexts (social and nonsocial). This measure carries forward all items from the SEQ-2.1. It has been psychometrically evaluated in a large sample of autistic children ages 2–12 years (Ausderau et al., 2014), demonstrating preliminary support for a complex factor structure consisting of four domain factors and additional method factors reflecting sensory modalities and social contexts. Though all quantitative items on the SEQ-3.0 were considered for inclusion in the current study, analogous items on the SEQ-2.1 and SEQ-3.0 were combined into single items for the purpose of cross-dataset analysis.

Measures of Putative Correlates

Cognitive Ability. Verbal, nonverbal, and full-scale intelligence quotients (VIQ, NVIQ, and FSIQ, respectively) were assessed using various tests based on the studies from which each child participant was drawn. The majority of children (86.6%) in the combined cohort completed a cognitive assessment; specifically, participants were administered a Wechsler IQ measure (45.9%; e.g., the WASI-II; Wechsler, 2011), the Mullen Scales of Early Learning (19.0%; Mullen, 1995), a version of the Differential Ability Scales (12.6%; e.g., the DAS-II; Elliott, 2007), or a version of the Leiter International Performance Scale (9.1%; e.g., Leiter-3; Roid et al., 2013). When possible, standard scores for VIQ/NVIQ/FSIQ were used, but in cases wherein developmental quotients (DQs; e.g., based on the Mullen) were reported and/or tests were administered to children outside of the measure's intended age range, DQ scores were utilized as reported or calculated using age-equivalent scores and chronological age information (i.e., DQ = $100 \times \frac{developmental\ age}{chronological\ age}$). In addition to dimensional IQ scores, we also defined a dichotomous "Intellectual Disability" variable, defined as a FSIQ score < 70 or NVIQ < 70 in cases where no FSIQ was available (individuals with DQ scores only were excluded from this measure).

Adaptive Behavior. Adaptive behavior in the current study was measured via summary scores from the Vineland Adaptive Behavior Scales (VABS; Sparrow, 2011), including the first, second, and third editions of the measure. When available, standard scores from the VABS Communication (COM) domain, Daily Living Skills (DLS) domain, Socialization (SOC) domain, and Adaptive Behavior Composite (ABC) were analyzed. The majority of scores in the current sample were derived from the Vineland-II (56.1%; Sparrow et al., 2005) and Vineland-3 (41.5%; Sparrow et al., 2016).

Core Autism Features. Core autism features were predominantly measured using caregiver-report questionnaires. As these measures are not interchangeable or measured on the

same scale, we chose to only examine the most commonly used single measures in our sample, namely the Social Responsiveness Scale–School Age (SRS [including both the first and second editions]; Constantino et al., 2003; Constantino & Gruber, 2012) and the Repetitive Behavior Scale–Revised (RBS-R; Bodfish et al., 2000). For individuals with the SRS, we examined total raw scores only, as the SRS is largely unidimensional, assessing autism features as a single construct (Sturm et al., 2017). For the RBS-R, we calculated subscale scores based on the factor structure demonstrated by Bishop et al. (2013), examining the repetitive sensory motor (RSM), self-injurious behavior (SIB), ritualistic/sameness behavior, and compulsive behavior subscales in the current study. Notably, in our combined cohort, the ritualistic/sameness and compulsive subscales of the RBS-R correlated very highly (Pearson r = .751); thus, the two scales were summed together (forming the "RSC" scale) in the current analysis of individual differences. The two-item restricted interests subscale was not examined due to its low reliability and poor content coverage of the larger trait domain of circumscribed interests (Nowell et al., 2021; Turner-Brown et al., 2011; Uljarević et al., 2021, 2023).

Co-occurring Psychiatric Symptoms. Co-occurring psychiatric symptoms were measured using a number of disorder-specific and transdiagnostic measures, although concordance across studies was somewhat more limited than with other clinical measures. In the current study, we opted to examine the broad trait domains of internalizing symptoms (INT), externalizing symptoms (EXT), total psychiatric symptoms (TOT), and features of attention deficit hyperactivity disorder (ADHD). The Achenbach System of Empirically Based Assessment (ASEBA; Achenbach, 2009) suite of measures, including the Child Behavior Checklist (CBCL) ages 1.5–5 and 6–18, was used in the majority of cases (75.7%). A minority of children had scores derived from a version of the Behavior Assessment System for Children

(BASC; Reynolds & Kamphaus, 2015) or the Conners 3rd Edition (Conners-3; used only as a measure of ADHD symptoms; Conners, 2008). Scores from all psychiatric symptom measures were analyzed on a common T-score metric (*M* = 50, *SD* = 10) to improve comparability across datasets.

Data Analysis

Sensory Subconstruct Refinement and Empirical Item Removal

Subscale item refinement was conducted in the calibration sample using an iterative process based on hierarchical item clustering with the ICLUST algorithm (Cooksey & Soutar, 2006; Revelle, 1978), as implemented in the *psych R* package (Revelle, 2022). The ICLUST algorithm was first applied to the inter-item polychoric correlations of a given scale (based on pairwise complete data), and the resulting cluster structure was examined. Items or sub-clusters that, when added to the main cluster, significantly reduced the cluster's coefficient beta by ≥ 0.1 were iteratively removed (Rosenthal et al., 2021; Sapey-Triomphe et al., 2018), and item pairs that were much more strongly related to each other than all other indicators of a single-modality subconstruct (i.e., those with outlier correlations based on the MAD-median rule; Wilcox & Rousselet, 2018) were combined into single items to eliminate local dependency. The clustering process was repeated iteratively after removing or merging items until (a) the resulting scale formed a single cluster of at least three items and (b) no further items/subclusters were flagged for removal or consolidation.

After item removal, the resulting scale was tested for unidimensionality and reliability by being fit to a unidimensional graded response model (GRM; Samejima, 1969), a type of IRT model, which was subsequently evaluated in terms of global model fit (unidimensionality) and marginal reliability. Models were fit using marginal maximum likelihood based on the Bock-

Aitkin EM algorithm (Bock & Aitkin, 1981), as implemented in the *mirt* R package (Chalmers, 2012). Missing data within each GRM were handled using full-information maximum likelihood estimation. For models with four or more items, global fit was assessed using the limited-information C_2 fit index (Cai & Monroe, 2014; Monroe & Cai, 2015) accompanied by C_2-based approximate fit indices, including the Tucker-Lewis index (TLI_{C2}; Cai et al., 2023), root mean square error of approximation ($RMSEA_{C2}$; Maydeu-Olivares & Joe, 2014), and standardized root-mean-square residual (SRMR; Maydeu-Olivares & Joe, 2014). For models with only three items, only the SRMR was calculated. Reliability was assessed using categorical coefficient omega (Green & Yang, 2009) and IRT-based marginal reliability (Samajima, 1994), and values of .70 or greater for both coefficients were judged to be acceptable for the current study. Adequate model fit of a scale was defined as $TLI_{C2} > 0.97$, $RMSEA_{C2} < .089$, and SRMR < .05 (Cai et al., 2023; Maydeu-Olivares & Joe, 2014), and for three-item scales, adequate fit was defined as SRMR < .033. In cases where model fit was poor, additional items were removed if their residual correlations indicated significant local misfit (i.e., approximately $r_{res} > 0.1$); this process was repeated until adequate model fit was achieved.

Bifactor Modeling of Sensory Constructs

First, to confirm the approximate simple structure of the data within each response pattern, we conducted exploratory graph analysis (EGA; based on the Golino et al., 2020 algorithm) on the inter-item polychoric correlations using the *EGAnet* R package (Golino & Christensen, 2020). This structure was confirmed by examining assignments of items to “communities” from the EGA procedure (based on the Walktrap algorithm; Christensen et al., 2023) to ensure that all items loaded with others from their same modality (with the exception of single-item subconstructs). Once the simple structure was confirmed, we fit the item-level data to

a bifactor GRM (Gibbons et al., 2007; Toland et al., 2017) using the *mirt* package's bifactor EM algorithm (Chalmers, 2012). Model fit was assessed using the limited-information M_2^* and M_2^*-based incremental fit indices (Maydeu-Olivares, 2013), with the same criteria for adequate fit as used for the unidimensional GRMs (i.e., $TLI_{M2} > 0.97$, $RMSEA_{M2} < .089$, and SRMR < .05, without substantial local misfit).

Supplemental Table S1

Participant demographics and broader characteristics for samples included in integrative data analysis

	UNC	VUMC1	UCSF	Syracuse	UCLA	Jefferson	Reading
Sample Size	104	181	35	55	67	93	37
Age (years)	6.77 ± 2.37 (104)	10.14 ± 3.48 (181)	10.21 ± 1.32 (35)	11.86 ± 3.28 (55)	13.75 ± 3.13 (67)	7.05 ± 1.34 (93)	8.68 ± 2.82 (37)
Female Sex	19 (18.3%)	25 (13.9%)	1 (2.9%)	14 (25.5%)	17 (25.4%)	9 (9.7%)	8 (21.6%)
**Ethnicity**							
Hispanic or Latino	0 (0%)	7 (3.9%)	0 (0%)	0 (0%)	15 (22.4%)	6 (6.5%)	0 (0%)
Not Hispanic or Latino	104 (100%)	150 (82.9%)	0 (0%)	52 (94.5%)	18 (26.9%)	86 (92.5%)	18 (48.6%)
Not Reported or Unknown	0 (0%)	24 (13.3%)	35 (100%)	3 (5.5%)	34 (50.7%)	1 (1.1%)	19 (51.4%)
**Race**							
White	0 (0%)	142 (78.5%)	0 (0%)	50 (90.9%)	18 (26.9%)	79 (84.9%)	16 (43.2%)
American Indian or Alaska Native	0 (0%)	3 (1.7%)	0 (0%)	0 (0%)	1 (1.5%)	0 (0%)	0 (0%)
Asian	0 (0%)	4 (2.2%)	0 (0%)	1 (1.8%)	3 (4.5%)	3 (3.2%)	0 (0%)
Black or African American	0 (0%)	11 (6.1%)	0 (0%)	0 (0%)	2 (3.0%)	7 (7.5%)	0 (0%)
Native Hawaiian or Other Pacific Islander	0 (0%)	0 (0%)	0 (0%)	0 (0%)	0 (0%)	0 (0%)	0 (0%)
More than One Race	0 (0%)	13 (7.2%)	0 (0%)	3 (5.5%)	10 (14.9%)	0 (0%)	2 (5.4%)
Not Reported or Unknown	104 (100%)	8 (4.4%)	35 (100%)	1 (1.8%)	33 (49.3%)	4 (4.3%)	19 (51.4%)
**Sensory Measures Administered**							
SP1/ SSP1	102 (98.1%)	172 (95.0%)	35 (100%)	53 (96.4%)	67 (100%)	92 (98.9%)	19 (51.4%)
SP2/ SSP2	0 (0%)	0 (0%)	0 (0%)	3 (5.5%)	0 (0%)	0 (0%)	18 (48.6%)
SEQ Version 2.1	42 (40.4%)	170 (93.9%)	0 (0%)	0 (0%)	0 (0%)	91 (97.8%)	0 (0%)
SEQ Version 3.0	59 (56.7%)	0 (0%)	0 (0%)	0 (0%)	0 (0%)	0 (0%)	0 (0%)
**Cognitive Scores**							
Intelligence Quotient (IQ)	51 (49.0%)	154 (85.1%)	34 (97.1%)	48 (87.3%)	65 (97.0%)	87 (93.5%)	35 (94.6%)
Developmental Quotient (DQ)	51 (49.0%)	18 (9.9%)	0 (0%)	0 (0%)	0 (0%)	0 (0%)	0 (0%)
Neither	2 (1.9%)	9 (5.0%)	1 (2.9%)	7 (12.7%)	2 (3.0%)	6 (6.5%)	2 (5.4%)
Full-scale IQ/DQ	60.4 ± 17.0 (49)	93.1 ± 26.2 (171)	98.1 ± 16.1 (34)	103.0 ± 12.8 (48)	104.6 ± 16.2 (65)	97.0 ± 19.1 (83)	106.6 ± 19.0 (28)
Verbal IQ/DQ	52.4 ± 27.6 (49)	90.8 ± 27.4 (172)	101.4 ± 21.2 (34)	99.3 ± 15.1 (48)	101.4 ± 17.8 (65)	97.4 ± 18.7 (81)	n.r.
Nonverbal IQ/DQ	72.4 ± 27.0 (98)	95.4 ± 24.8 (170)	102.1 ± 15.6 (34)	106.4 ± 14.0 (48)	107.4 ± 17.1 (65)	98.1 ± 18.1 (84)	n.r.
**Adaptive Functioning**							
Vineland ABC	59.8 ± 16.0 (102)	n.r.	n.r.	73.7 ± 12.3 (29)	77.6 ± 14.7 (59)	78.3 ± 11.0 (88)	n.r.
Vineland COM	69.5 ± 22.6 (102)	n.r.	n.r.	79.1 ± 16.0 (29)	79.1 ± 15.3 (59)	85.1 ± 13.5 (92)	n.r.
Vineland DLS	56.6 ± 17.2 (100)	n.r.	n.r.	79.9 ± 16.7 (29)	83.2 ± 17.3 (59)	82.2 ± 12.2 (92)	n.r.
Vineland SOC	65.4 ± 13.5 (101)	n.r.	n.r.	67.1 ± 12.1 (29)	75.9 ± 17.3 (59)	75.8 ± 13.3 (92)	n.r.
**Psychiatric Symptoms**							
TOTAL	60.1 ± 8.7 (71)	n.r.	n.r.	68.9 ± 10.7 (51)	64.6 ± 7.8 (23)	n.r.	n.r.
INT	57.4 ± 10.0 (71)	n.r.	n.r.	63.8 ± 17.2 (51)	62.2 ± 10.8 (23)	n.r.	n.r.
EXT	54.5 ± 9.0 (71)	n.r.	n.r.	57.5 ± 10.5 (51)	57.0 ± 10.6 (23)	n.r.	n.r.

Values are presented as *n* (%) for categorical variables and *M* ± *SD (n)* for continuous variables. Cognitive scores (derived from measures including various Wechsler IQ scales (Wechsler, 2011), the Mullen Scales of Early Learning (Mullen, 1995), a version of the Differential Ability Scales (Elliott, 2007), or a version of the Leiter International Performance Scale (Roid et al., 2013) in the majority of cases) and adaptive functioning scores (derived from the Vineland Adaptive Behavior Scales; Sparrow, 2011) are presented as standard scores (M = 100, SD = 15). Psychiatric symptom scores (derived from the Achenbach System of Empirically Based Assessment (Achenbach, 2009) or a version of the Behavior Assessment System for Children (Reynolds & Kamphaus, 2015)) are presented as T-scores (M = 50, SD = 10). Values of "n.r." indicate that a score was not reported in a given study. SP = Sensory Profile; SSP = Short Sensory Profile; SEQ = Sensory Experiences Questionnaire; ABC = Adaptive Behavior Composite; COM = Communication; DLS = Daily Living Skills; SOC = Socialization; INT = Internalizing Symptoms; EXT = Externalizing Symptoms; UNC = University of North Carolina; VUMC = Vanderbilt University Medical Center; UCSF = University of California San Francisco; UCLA = University of California Los Angeles; Jefferson = Thomas Jefferson University [some participants also recruited from Albert Einstein College of Medicine]; Reading = University of Reading [some participants also recruited from Mount Sinai School of Medicine].

Supplemental Table S1 (Continued)

	KKI	VUMC2	NDAR	SEP	SPARK	Full Sample
Sample Size	47	114	741	1285	1107	3866
Age (years)	10.47 ± 1.34 (47)	11.14 ± 3.78 (114)	9.33 ± 3.95 (741)	7.78 ± 2.65 (1285)	7.58 ± 3.04 (1107)	8.41 ± 3.36 (3866)
Female Sex	5 (10.6%)	22 (19.3%)	165 (22.3%)	232 (18.1%)	191 (17.3%)	793 (20.5%)
**Ethnicity**						
Hispanic or Latino	0 (0%)	9 (7.9%)	59 (8.0%)	0 (0%)	188 (17.0%)	284 (7.3%)
Not Hispanic or Latino	0 (0%)	89 (78.1%)	518 (69.9%)	3 (0.2%)	919 (83.0%)	1957 (50.6%)
Not Reported or Unknown	47 (100%)	16 (14.0%)	164 (22.1%)	1282 (99.8%)	0 (0%)	1625 (42.0%)
**Race**						
White	35 (74.5%)	83 (72.8%)	501 (67.6%)	1091 (84.9%)	904 (81.7%)	2919 (75.5%)
American Indian or Alaska Native	0 (0%)	0 (0%)	0 (0%)	7 (0.5%)	30 (2.7%)	41 (1.1%)
Asian	0 (0%)	2 (1.8%)	35 (4.7%)	16 (1.2%)	42 (3.8%)	106 (2.7%)
Black or African American	7 (14.9%)	6 (5.3%)	36 (4.9%)	37 (2.9%)	3 (0.3%)	109 (2.8%)
Native Hawaiian or Other Pacific Islander	0 (0%)	0 (0%)	2 (0.3%)	2 (0.2%)	0 (0%)	4 (0.1%)
More than One Race	5 (10.6%)	8 (7.0%)	48 (6.5%)	101 (7.9%)	89 (8.0%)	279 (7.2%)
Not Reported or Unknown	0 (0%)	15 (13.2%)	119 (16.1%)	31 (2.4%)	39 (3.5%)	408 (10.6%)
**Sensory Measures Administered**						
SP1/ SSP1	0 (0%)	114 (100%)	466 (62.9%)	453 (35.3%)	0 (0%)	1573 (40.0%)
SP2/ SSP2	0 (0%)	0 (0%)	272 (36.7%)	0 (0%)	0 (0%)	293 (7.6%)
SEQ Version 2.1	0 (0%)	101 (88.6%)	29 (3.9%)	0 (0%)	0 (0%)	433 (11.2%)
SEQ Version 3.0	47 (100%)	0 (0%)	0 (0%)	1285 (100%)	1107 (100%)	2498 (64.6%)
**Cognitive Scores**						
Intelligence Quotient (IQ)	45 (95.7%)	100 (87.7%)	386 (52.1%)	3 (0.2%)	34 (3.1%)	1042 (27.0%)
Developmental Quotient (DQ)	0 (0%)	0 (0%)	161 (21.7%)	1 (0.1%)	16 (1.4%)	247 (6.4%)
Neither	2 (4.3%)	14 (12.3%)	194 (26.2%)	1281 (99.7%)	1057 (95.5%)	2577 (66.7%)
Full-scale IQ/DQ	97.9 ± 15.6 (47)	n.r.	91.1 ± 25.0 (460)	n.r.	74.8 ± 26.4 (42)	92.1 ± 24.5 (1028)
Verbal IQ/DQ	98.9 ± 16 (47)	n.r.	86.0 ± 29.1 (512)	n.r.	85.6 ± 26.9 (29)	88.8 ± 27.9 (1038)
Nonverbal IQ/DQ	103.2 ± 15.2 (47)	105.9 ± 18.1 (100)	88.4 ± 28.0 (513)	73.7 ± 40.3 (3)	91.5 ± 25.9 (31)	93.0 ± 26.0 (1193)
**Adaptive Functioning**						
Vineland ABC	n.r.	n.r.	67.7 ± 16.0 (602)	55.5 ± 29.1 (4)	71.2 ± 15.9 (588)	69.7 ± 16.1 (1472)
Vineland COM	n.r.	78.5 ± 13.5 (111)	72.0 ± 18.7 (605)	60.3 ± 35.1 (4)	71.6 ± 21.1 (588)	73.3 ± 19.6 (1590)
Vineland DLS	n.r.	81.4 ± 17.6 (111)	68.0 ± 19.2 (466)	54.0 ± 28.4 (4)	73.8 ± 17.9 (588)	72.3 ± 19.1 (1449)
Vineland SOC	n.r.	71.6 ± 13.2 (111)	67.4 ± 16.7 (605)	62.0 ± 24.1 (4)	68.7 ± 18.8 (588)	68.8 ± 17.1 (1589)
**Psychiatric Symptoms**						
TOTAL	n.r.	69.5 ± 12.9 (102)	62.2 ± 9.4 (420)	54.8 ± 5.6 (4)	n.r.	63.6 ± 10.4 (671)
INT	n.r.	58.7 ± 11.6 (102)	60.1 ± 11.6 (514)	51.3 ± 12.1 (4)	n.r.	59.9 ± 11.9 (765)
EXT	n.r.	61.3 ± 14.4 (102)	55.7 ± 12.1 (515)	51.3 ± 7.8 (4)	n.r.	56.5 ± 12.2 (766)

Values are presented as *n* (%) for categorical variables and *M* ± *SD (n)* for continuous variables. Cognitive scores (derived from measures including various Wechsler IQ scales (Wechsler, 2011), the Mullen Scales of Early Learning (Mullen, 1995), a version of the Differential Ability Scales (Elliott, 2007), or a version of the Leiter International Performance Scale (Roid et al., 2013) in the majority of cases) and adaptive functioning scores (derived from the Vineland Adaptive Behavior Scales; Sparrow, 2011) are presented as standard scores (*M* = 100, *SD* = 15). Psychiatric symptom scores (derived from the Achenbach System of Empirically Based Assessment (Achenbach, 2009) or a version of the Behavior Assessment System for Children (Reynolds & Kamphaus, 2015)) are presented as T-scores (*M* = 50, *SD* = 10). Values of "n.r." indicate that a score was not reported in a given study. SP = Sensory Profile; SSP = Short Sensory Profile; SEQ = Sensory Experiences Questionnaire; ABC = Adaptive Behavior Composite; COM = Communication; DLS = Daily Living Skills; SOC = Socialization; INT = Internalizing Symptoms; EXT = Externalizing Symptoms; KKI = Kennedy-Krieger Institute; VUMC = Vanderbilt University Medical Center; NDAR = National Database for Autism Research; SEP = Sensory Experiences Project; SPARK = Simons Powering Autism Research for Knowledge (RM0035Woynaroski).

Supplemental Table S2

Relevant sensory questionnaire items organized by subconstruct (Page 1 of 3)

Item(s)	Summary of Item Content[a]	Included	%Missing (Calibration/Full)
**Auditory HYPER**			
SP1 Q1/SP2 Q1	Negative response to unexpected/loud noises	Yes*	12.2% / 55.7%
SP1 Q2/SP2 Q2	Covers ears with hands to protect against sound	Yes*	12.3% / 55.7%
SP1 Q3/SP2 Q3	Trouble completing tasks with TV/music/radio in background	Yes	1.1% / 52.4%
SP1 Q4/SP2 Q4	Trouble functioning/focusing with significant background noise	Yes	0.3% / 52.0%
SP1 Q5/SP2 Q5	Difficulty working/being productive with background noise	Yes	12.7% / 55.9%
SEQ2 Q1/SEQ3 Q1	Easily startled by unexpected/loud noises	Yes*	22.4% / 24.3%
SEQ2 Q6/SEQ3 Q10	Startle response/covers ears due to loud conversations/singing	No	22.3% / 24.3%
SEQ2 Q5/SEQ3 Q3	Notices environmental sounds (planes, trains, thunder) before others	No	22.3% / 24.2%
SEQ3 Q2	Noise making/humming to tune out or avoid sounds	No	57.4% / 35.4%
SEQ3 Q9	Bothered by everyday sounds (dishwasher, radio, etc.) that don't bother others	Yes*	57.4% / 35.5%
**Visual HYPER**			
SP1 Q9/SP2 Q9	Prefers to be/play/work in low-light/dark settings	No	18.7% / 62.8%
SP1 Q10/SP2 Q15	Bothered by and/or avoidance of bright lights	Yes	18.2% / 62.6%
SP1 Q14/SP2 Q13	Bothered by bright lights more than/for longer than other children	Yes	13.3% / 56.2%
SEQ2 Q8/SEQ3 Q15	Disturbed by bright lights inside or brightness outside	Yes	22.5% / 24.5%
SEQ2 Q11/SEQ3 Q18	Avoidance of gaze towards parent's face during social games/play	No	22.5% / 24.5%
SP1 Q11	Happy to be in the dark	No	29.5% / 66.1%
SP1 Q15	Squinting/covering of eyes to protect from light	Yes	24.4% / 59.4%
SP1 Q96	Avoids eye contact	No	29.4% / 66.1%
SEQ3 Q33	Visual distraction due to objects in motion around him/her	No	57.4% / 36.0%
**Tactile HYPER**			
SP1 Q30/SP2 Q16/ SEQ2 Q15/SEQ3 Q49	Distress shown during grooming (crying/fighting)	Yes*	0.3% / 0.9%
SP1 Q34/SP2 Q17	Becomes irritated by wearing socks or shoes	Yes	18.3% / 62.6%
SP1 Q36/SP2 Q18	Emotional/aggressive reaction to being touched	Yes*	13.7% / 56.2%
SP1 Q39/SP2 Q20	Rubs/scratches part of body that has been touched	Yes	14.0% / 56.2%
SEQ2 Q14/SEQ3 Q36	Dislike of being held or cuddled	No	22.6% / 24.9%
SEQ2 Q16/SEQ3 Q38	Avoidance of touching certain textures (like fuzzy/squishy toys) or playing with messy materials (like sand/lotion)	Yes*	22.6% / 24.9%
SEQ2 Q17/SEQ3 Q51	Aversion towards being touched by another person	Yes	22.6% / 25.1%
SEQ2 Q18/SEQ3 Q40	Trouble adjusting to bath/shower water temperature or aversion to being in water	No	22.7% / 25.0%
SEQ2 Q20/SEQ3 Q52	Dislikes being tickled	No	22.6% / 25.1%
SP1 Q29	Avoidance of getting messy (playing with sand, glue, etc.)	Yes	29.5% / 66.1%
SP1 Q32	Showing discomfort (crying/fighting) towards dental work/toothbrushing	Yes	29.5% / 66.1%
SP1 Q33	Sensitivity to certain fabrics (particularly certain clothes/bedsheets)	Yes*	29.6% / 66.1%
SP1 Q35	Avoidance of going barefoot, especially in sand/grass	No	24.6% / 59.4%
SP1 Q37	Withdrawal from splashing water	No	24.6% / 59.4%
SEQ3 Q44	Aversion/sensitivity towards sticky materials (like tape, band-aids)	Yes	57.4% / 36.1%
SEQ3 Q46	Bothered by tags or seams on clothing	Yes	57.4% / 36.1%
SEQ3 Q47	Bothered by tight clothing (like turtlenecks, stocking caps)	Yes	57.4% / 36.1%
SEQ3 Q54	Negative reaction to haircuts	Yes	57.4% / 36.1%
**Olfactory HYPER**			
SEQ3 Q61	Aversion/sensitivity to the way people smell	Yes	57.4% / 36.2%
SEQ3 Q66	Noticing smells before other people do	Yes	57.4% / 36.3%
SEQ3 Q73	Gagging in response to certain smells	Yes	57.4% / 36.3%
**Gustatory HYPER**			
SP1 Q54/SP2 Q43	Easily gags due to certain food textures/utensils in mouth	No	18.1% / 62.6%
SP1 Q55/SP2 Q44	Rejects/avoids certain food tastes/smells typically found in children's diet	Yes	12.9% / 55.8%
SP1 Q56/SP2 Q45	Will only eat certain tastes (like sweet, salty, etc.)	Yes	13.6% / 56.1%
SP1 Q57/SP2 Q46	Limits self to particular food textures/temperatures	No	13.5% / 56.0%
SP1 Q58/SP2 Q47	Picky eater, especially about food textures	No	13.0% / 55.9%
SEQ2 Q22/SEQ3 Q59	Refuses to try new foods or avoids certain food tastes/smells/textures	Yes	22.6% / 25.1%
SEQ3 Q63	Seems to be a picky eater	No	57.4% / 36.2%
SEQ3 Q70	Able to taste the difference in subtle modifications to food (brand swap, recipe change)	Yes	57.4% / 36.3%
SEQ3 Q72	Refusal to eat food that he/she usually likes if it is mixed with another food	No	57.4% / 36.2%

Table continues on following page

Supplemental Table S2 (Continued; Page 2 of 3)

Item(s)	Summary of Item Content[a]	Included	%Missing
**Movement HYPER**			
SEQ2 Q29/ SEQ3 Q77	Seems uneasy/becomes dizzy when moving (like on a swing/rocking chair)	No	22.6% / 25.2%
SP1 Q18	Becomes anxious/distressed when his/her feet leave the ground	Yes	24.5% / 59.4%
SP1 Q19	Dislikes activities where his/her head is upside down (like somersaults, roughhousing)	Yes	24.5% / 59.5%
SP1 Q20	Avoids playground equipment/moving toys (like swings, merry-go-rounds)	Yes	29.3% / 66.0%
SP1 Q21	Dislikes riding in a car	No	29.4% / 66.1%
SP1 Q23	Becomes disoriented (falls or dizziness) after bending over sink/table	No	29.6% / 66.1%
SP1 Q77	Fears falling or heights	No	24.5% / 59.4%
SP1 Q78	Avoids climbing/jumping or bumpy/uneven ground	No	29.4% / 66.3%
SEQ3 Q81	Avoids riding on escalators, elevators, or other moving surfaces	No	57.4% / 36.3%
SEQ3 Q83	Negatively reacts to being moved quickly by a familiar adult	Yes	57.4% / 36.3%
**Auditory HYPO**			
SP1 Q6/SP2 Q6	Appears to tune you out/not hear what you say/ignore you	Yes	0.3% / 52.2%
SP1 Q7/SP2 Q7	Doesn't hear/respond when name is called although hearing is OK	Yes	12.6% / 55.8%
SEQ2 Q4/SEQ3 Q4	Seems to ignore or tune-out loud noises (for example, no reaction when alarm goes off)	No	22.3% / 24.3%
SEQ2 Q3/SEQ3 Q8	Ignores you when you call his/her name	Yes	22.3% / 24.3%
SEQ3 Q14	Trouble distinguishing between different types of sound	No	57.4% / 35.4%
**Visual HYPO**			
SP1 Q99/SEQ2 Q12/SEQ3 Q31	Doesn't notice/ignores new or different people when they enter the room	No	22.2% / 12.8%
SEQ2 Q10/SEQ3 Q22 /SEQ3 Q23	Slowness in noticing new objects/toys entering the room even when placed near him/her	Yes	22.5% / 24.5%
SEQ3 Q34	Trouble distinguishing between different types of visual sensations	No	57.4% / 36.1%
**Tactile HYPO**			
SP1 Q42/SP2 Q23/SP2 Q24	Unaware of pain or temperature changes	Yes	18.2% / 25.1%
SP1 Q46/SP2 Q26	Doesn't seem to notice (oblivious to) when face or hands are messy	No	12.8% / 25.0%
SEQ2 Q19/SEQ3 Q53	Slow to react to pain (for example, not being bothered by scrapes, cuts, falls)	Yes	22.6% / 62.6%
SEQ2 Q21/SEQ3 Q43	Ignores/doesn't notice you when you tap him/her on shoulder for attention	No	22.6% / 58.7%
SP1 Q31	Prefers long-sleeved clothing in warm weather or short sleeves in cold weather	No	24.9% / 59.5%
SP1 Q43	Seems not to notice when someone touches his/her arm or back	No	29.4% / 66.1%
SP1 Q53	Leaves clothing twisted on body	No	24.5% / 59.4%
SEQ3 Q56	Unawareness of extreme temperatures (hot or cold)	Yes	57.4% / 36.1%
SEQ3 Q58	Trouble distinguishing between different types of touch sensations	No	57.4% / 36.2%
**Olfactory HYPO**			
SP1 Q125	Does not seem to smell strong odors	Yes	29.7% / 66.2%
SEQ3 Q69	Unawareness of strong or unpleasant smells that most people notice	Yes	57.4% / 36.2%
**Gustatory HYPO**			
SEQ3 Q74	Trouble distinguishing between different types of tastes or flavors	Yes	57.4% / 36.3%
**Auditory SEEK**			
SP1 Q8/SP2 Q8	Enjoys strange noises or seeks to make noise for noise's sake/fun	No	12.7% / 55.7%
SEQ2 Q36a/SEQ3 Q7	Extreme fascination with sounds	Yes	36.0% / 28.8%
SEQ3 Q11	Fascination/excitement towards the sound of others singing	No	57.4% / 35.4%
**Visual SEEK**			
SP1 Q97/SP2 Q80/SP2 Q81	Stares intensely at objects/people	Yes	18.0% / 62.5%
SEQ2 Q9/SEQ3 Q17	Stares at lights/objects that spin or move	Yes	22.5% / 24.6%
SEQ2 Q36b/SEQ3 Q21	Extreme fascination with lights	No	36.0% / 28.9%
SP1 Q16	Looks carefully/intensely at objects/people (staring)	No	29.3% / 66.1%
SEQ3 Q16	Fascination with his/her own reflection in another person's eyes	No	57.4% / 35.7%
SEQ3 Q19	Fascination/excitement due to the visual effect of water falling, swirling, or pouring	Yes	57.4% / 35.8%
SEQ3 Q25	Looking at objects using side vision or out of the corner of his/her eyes	No	57.4% / 36.0%
SEQ3 Q27	Watching objects that he/she moves rapidly in front of his/her eyes	Yes	57.4% / 36.0%
SEQ3 Q28	Fascination/excitement due to his/her reflection in shiny surfaces (like mirrors, sunglasses)	No	57.4% / 36.0%
SEQ3 Q29	Looking at angles or corners of objects (like a table leg)	Yes	57.4% / 36.0%
SEQ3 Q30	Fascination/excitement due to flickering or scrolling motions on a computer screen/TV	Yes	57.4% / 36.0%
SEQ3 Q32	Watching his/her hands or fingers move/flicker in front of the eyes	No	57.4% / 36.0%
**Tactile SEEK**			
SP1 Q40/SP1 Q45/SP2 Q21/SP2 Q25	Touches people and objects to the point of irritating others, more so than same-aged children	Yes	29.6% / 66.1%
SP1 Q41/SP2 Q22	Displays unusual need for touching certain toys, surfaces, or textures	No	24.5% / 59.4%
SEQ2 Q36f/SEQ3 Q45	Extreme fascination with touch	Yes	29.6% / 66.1%
SEQ2 Q36e/SEQ3 Q55	Extreme fascination with textures of surfaces, objects, or clothing fabrics	No	29.6% / 66.1%
SP1 Q44	Avoids wearing shoes; loves being barefoot	No	24.5% / 59.4%
SEQ3 Q37	Craves intense or firm touch (like firm hugs, squeezes, back scratches)	Yes	29.6% / 66.1%
SEQ3 Q39	Enjoys air blowing from fans or wind on his/her face	No	29.6% / 66.1%
SEQ3 Q41	Craves being tickled	No	24.5% / 59.4%
SEQ3 Q50	Touches/rubs certain surfaces/materials (grooves, ridges, smooth surfaces)	Yes	29.6% / 66.1%
SEQ3 Q57	Rubs wet substances (lotion, water, soap) onto a tabletop or another smooth surface	No	29.6% / 66.1%

Table continues on following page

Supplemental Table S2 (Continued; Page 3 of 3)

Item(s)	Summary of Item Content[a]	Included	%Missing
**Olfactory SEEK**			
SP1 Q59/SP2 Q48	Routinely smells non-food objects	No	18.3% / 62.7%
SEQ2 Q23/SEQ3 Q60	Smells objects or toys during play or other activities	No	22.6% / 25.1%
SEQ2 Q36c/SEQ3 Q67	Extreme fascination with particular smells	Yes	36.0% / 29.5%
SP1 Q60	Strongly prefers certain smells	No	29.7% / 66.3%
SP1 Q124	Deliberately smells objects	No	29.7% / 66.2%
**Gustatory SEEK**			
SP1 Q61/SP2 Q49	Strongly prefers certain tastes	No	5.9% / 58.9%
SEQ2 Q36d/SEQ3 Q65	Extreme fascination with particular tastes	No	36.0% / 29.5%
SP1 Q62	Craves certain foods	No	29.5% / 66.2%
SP1 Q63	Seeks out certain tastes/smells	No	29.8% / 66.3%
SEQ3 Q64	Craves foods with a strong taste or flavor (like spicy, sour, or bitter foods)	Yes	57.4% / 36.2%
**Oral Tactile SEEK**			
SEQ2 Q25/SEQ3 Q62	Puts objects, toys, or other non-food items in his/her mouth to lick, suck, chew, or explore	Yes	22.6% / 25.1%
SP1 Q64	Chews or licks nonfood objects	Yes	29.5% / 66.2%
SP1 Q65	Mouths objects (like pencils, hands)	Yes	29.5% / 66.1%
SEQ3 Q68	Mouths, sucks, or chews on his/her own hands or fingers	No	57.4% / 36.3%
SEQ3 Q71	Licks non-food objects	Yes	57.4% / 36.2%
**Movement SEEK**			
SP1 Q24/SP1 Q25/SP2 Q27	Pursual of all kinds of movement activities which interferes with daily routines (can't sit still)	Yes	0.6% / 52.0%
SP1 Q27/SP1 Q28/SP2 Q28	Unconscious rocking possibly in desk/chair/on floor/while standing	No	6.9% / 25.2%
SP1 Q83/SP2 Q32	Seeks out opportunities to fall without regard for personal safety	No	18.6% / 59.1%
SP1 Q84/SP2 Q60	Appears to enjoy falling	Yes	18.5% / 24.4%
SEQ2 Q27/SEQ3 Q76	Likes to jump up/down, rock back/forth, or spin in circles	Yes	22.6% / 62.8%
SEQ2 Q28/SEQ3 Q78	Seeks out physical rough-housing play (like being tossed in air or spun)	No	22.6% / 62.7%
SEQ2 Q30/SEQ3 Q80	Flaps his/her arms or hands repeatedly, particularly when excited	No	22.6% / 25.2%
SP1 Q26	Twirls/spins self frequently throughout the day (enjoys dizziness)	Yes	29.5% / 66.1%
SEQ3 Q79	Enjoys being upside-down	No	57.4% / 36.5%
SEQ3 Q85	Adopts unusual postures with his/her hands, fingers, or arms	No	57.4% / 36.3%

Items included in the final operationalization of a given subconstruct (including single-item indicators) are shaded gray in the table. For Auditory HYPER and Tactile HYPER (in which both unidimensional and bifactor solutions were examined due to the presence of a viable bifactor model for the single-modality construct), the items that were included in the unidimensional composite score are marked with a *. HYPER = hyperreactivity; HYPO = hyporeactivity; SEEK = sensory seeking; SP1 = Sensory Profile 1 (Dunn, 1999); SP2 = Sensory Profile 2 (Dunn, 2014); SEQ2 = Sensory Experiences Questionnaire, version 2.1 (Baranek, 1999); SEQ3 = Sensory Experiences Questionnaire, version 3.0 (Ausderau et al., 2014; Baranek, 2009).

[a] Item content summaries have been adapted with permission from original measures with permission from the original copyright holders. To access the verbatim item content, interested parties are encouraged to contact the copyright holders directly (Grace Baranek [SEQ2/SEQ3] and NCS Pearson, Inc. [SP1/SP2]).

Supplemental Table S3

Specifications of hierarchical Bayesian integrative data analysis models

	Continuous Outcome Model	Binary Outcome Model
**Prior and Likelihood Specifications**<br><br>Outcome variables are standardized (M = 0, SD = 1)	$y_i \sim \mathrm{t}(\mu, \sigma, \nu)$<br>$\mu_i = \beta_{0,id} + \beta_{1,id} x_i$<br>$\begin{bmatrix}\beta_{0,id} \\ \beta_{1,id}\end{bmatrix} = \mathrm{MVNormal}\left(\begin{bmatrix}\beta_0 \\ \beta_1\end{bmatrix}, \Sigma\right)$<br>$\Sigma = \begin{pmatrix}\sigma_{\beta_0} & 0 \\ 0 & \sigma_{\beta_1}\end{pmatrix} \Omega \begin{pmatrix}\sigma_{\beta_0} & 0 \\ 0 & \sigma_{\beta_1}\end{pmatrix}$<br>$\beta_0 \sim \mathrm{t}_3(0, 2.5)$<br>$\beta_1 \sim \mathrm{Normal}(0, 0.5)$<br>$\nu \sim \mathrm{Gamma}(2, 0.1)_{lb=1}$<br>$\sigma_{\beta_0} \sim \text{Half-}\mathrm{t}_3(0, 2.5)$<br>$\sigma_{\beta_1} \sim \text{Half-}\mathrm{t}_3(0, 2.5)$<br>$\log(\sigma) \sim \mathrm{t}_3(0, 2.5)$<br>$\Omega \sim \mathrm{LKJcorr}(2)$	$y_i \sim \mathrm{t}(\mu, \sigma, \nu)$<br>$\mu_i = \beta_{0,id} + \beta_{1,id} x_i$<br>$\begin{bmatrix}\beta_{0,id} \\ \beta_{1,id}\end{bmatrix} = \mathrm{MVNormal}\left(\begin{bmatrix}\beta_0 \\ \beta_1\end{bmatrix}, \Sigma\right)$<br>$\Sigma = \begin{pmatrix}\sigma_{\beta_0} & 0 \\ 0 & \sigma_{\beta_1}\end{pmatrix} \Omega \begin{pmatrix}\sigma_{\beta_0} & 0 \\ 0 & \sigma_{\beta_1}\end{pmatrix}$<br>$\beta_0 \sim \mathrm{t}_3(0, 2.5)$<br>$\beta_1 \sim \mathrm{Normal}(0, 1)$<br>$\nu \sim \mathrm{Gamma}(2, 0.1)_{lb=1}$<br>$\sigma_{\beta_0} \sim \text{Half-}\mathrm{t}_3(0, 2.5)$<br>$\sigma_{\beta_1} \sim \text{Half-}\mathrm{t}_3(0, 2.5)$<br>$\log(\sigma_{x=0}) \sim \mathrm{Normal}(0, 1)$<br>$\log(\sigma_{x=1}) \sim \mathrm{Normal}(0, 1)$<br>$\Omega \sim \mathrm{LKJcorr}(2)$
**Effect Size Parameter**	$r = \frac{\beta_1}{\sqrt{\beta_1^2 + 1}}$<br><br>**ROPE** ($\mathcal{H}_0$)**:** $r = [-0.1, 0.1]$<br><br>**Prior scale:** ~50% density in r = [-0.32, 0.32]<br>~95% density in r = [-0.70, 0.70]<br>Prior $P(\mathcal{H}_0) \approx 16\%$	$d = \frac{\beta_1}{\sqrt{\frac{\sigma_{x=0}^2 + \sigma_{x=1}^2}{2}}}$<br><br>**ROPE** ($\mathcal{H}_0$)**:** $d = [-0.2, 0.2]$<br><br>**Prior scale:** ~50% density in d = [-0.45, 0.45]<br>~95% density in d = [-2.50 2.50]<br>Prior $P(\mathcal{H}_0) \approx 15\%$
**Heterogeneity Parameters**	$\tau^2 = \sigma_{\beta_1}^2$<br>$I^2 = 100 \times \frac{\tau^2}{\tau^2 + \mathrm{VAR}(\beta_1)}$<br><br>$\mathrm{ICC} = \frac{\tau^2 + \sigma_{\beta_0}^2}{\tau^2 + \sigma_{\beta_0}^2 + \sigma^2}$	$\tau^2 = \sigma_{\beta_1}^2$<br>$I^2 = 100 \times \frac{\tau^2}{\tau^2 + \mathrm{VAR}(\beta_1)}$<br><br>$\mathrm{ICC} = \frac{\tau^2 + \sigma_{\beta_0}^2}{\tau^2 + \sigma_{\beta_0}^2 + \sqrt{\frac{\sigma_{x=0}^2 + \sigma_{x=1}^2}{2}}}$
**MCMC Specifications**	Markov Chains: 10<br>Iterations Per Chain: 2,500<br>Warmup Iterations: 1,000<br>10 datasets (1 per plausible value)	Markov Chains: 10<br>Iterations Per Chain: 2,500<br>Warmup Iterations: 1,000<br>10 datasets (1 per plausible value)

Integrative data analysis models were fit as hierarchical Student-*t* regressions with random slope and intercept terms by data source (i.e., study sample). All outcomes were standardized prior to fitting. ROPE = Region of Practical Equivalence; MCMC = Markov Chain Monte Carlo.

Supplemental Table S4

Final bifactor structure of auditory hyperreactivity (HYPER) items

Item	G	S_1	h^2	*I-ECV*
SP1 Q1/SP2 Q1	0.886	—	0.786	1.000
SP1 Q2/SP2 Q2	0.852	—	0.726	1.000
SP1 Q3/SP2 Q3	0.329	0.842	0.817	0.132
SP1 Q4/SP2 Q4	0.451	0.620	0.588	0.346
SP1 Q5/SP2 Q5	0.455	0.520	0.478	0.434
SEQ2 Q1/SEQ3 Q1	0.878	—	0.771	1.000
SEQ3 Q9	0.726	0.220	0.575	0.916
**Bifactor Coefficients**				
ω_T/ω_S	0.945	0.874		
ω_H/ω_{HS}	0.759	0.491		
ECV_G	0.702	0.425		
ECV_{SS}	—	0.575		

Factor loadings are derived from full-information maximum likelihood confirmatory bifactor analysis, equivalent to a bifactor graded response model. All loadings are fully standardized, based on latent traits with means of 0 and standard deviations of 1 in the (autistic) population. G and S_1 columns represent the general and specific factors, respectively. For this model, the general factor represents the overall Auditory hyperreactivity (HYPER) construct, whereas the specific factor is interpreted as "auditory distractibility and intolerance of background noise." SP = Sensory Profile; SEQ = Sensory Experiences Questionnaire; h^2 = communality; *I-ECV* = item explained common variance; ECV_G = General factor explained common variance; ω_T = Coefficient omega total (total score reliability; ≥ 0.8 indicates high reliability); ω_S = Coefficient omega subscale (subscale reliability; > 0.8 indicates high reliability); ω_H = Coefficient omega hierarchical (general factor saturation; $\omega_H \geq 0.8$ or combination of $\omega_H \geq 0.7$ and $ECV_G \geq 0.60$ indicates strong general factor); ω_{HS} = Coefficient omega hierarchical subscale (specific factor saturation; ≥ 0.20/0.25 support the added value of a specific factor under conditions of high/low reliability, respectively); ECV_{SS} = Specific factor explained common variance for a subscale (≥ 0.30/0.45 support the added value of a specific factor under conditions of high/low reliability, respectively).

Supplemental Table S5

Final bifactor structure of tactile hyperreactivity (HYPER) items

Item	G	S_1	S_2	S_3	S_4	h^2	*I-ECV*
SP1 Q30/SP2 Q16/ SEQ2 Q15/SEQ3 Q49	0.595	—	—	0.649	—	0.775	0.457
SP1 Q34/SP2 Q17	0.703	—	0.266	—	—	0.565	0.875
SP1 Q36/SP2 Q18	0.713	—	—	—	0.575	0.840	0.606
SP1 Q39/SP2 Q20	0.654	—	—	—	0.362	0.559	0.765
SEQ2 Q16/SEQ3 Q38	0.613	0.610	—	—	—	0.748	0.502
SEQ2 Q17/SEQ3 Q51	0.596	—	—	—	0.511	0.616	0.577
SP1 Q29	0.399	0.777	—	—	—	0.763	0.209
SP1 Q32	0.565	—	—	0.490	—	0.560	0.571
SP1 Q33	0.758	—	0.449	—	—	0.777	0.740
SEQ3 Q44	0.540	0.384	—	—	—	0.439	0.664
SEQ3 Q46	0.633	—	0.678	—	—	0.860	0.465
SEQ3 Q47	0.653	—	0.447	—	—	0.626	0.681
SEQ3 Q54	0.390	—	—	0.565	—	0.472	0.323
**Bifactor Coefficients**							
ω_T/ω_S	0.946	0.823	0.886	0.789	0.839		
ω_H/ω_{HS}	0.789	0.450	0.270	0.420	0.283		
ECV_G	0.563	0.424	0.670	0.457	0.641		
ECV_{SS}	—	0.576	0.330	0.543	0.359		

Factor loadings are derived from full-information maximum likelihood confirmatory bifactor analysis, equivalent to a bifactor graded response model. All loadings are fully standardized, based on latent traits with means of 0 and standard deviations of 1 in the (autistic) population. G and S_{1-4} columns represent the general and specific factors, respectively. For this model, the general factor represents the overall Tactile hyperreactivity (HYPER) construct, specific factor S_1 is interpreted as "avoidance of messy/sticky textures," specific factor S_2 is interpreted as "distress/irritation caused by clothing," specific factor S_3 is interpreted as "difficulty/distress with grooming," and specific factor S_4 is interpreted as "aversive/defensive reactions to (social) touch." SP = Sensory Profile; SEQ = Sensory Experiences Questionnaire; h^2 = communality; *I-ECV* = item explained common variance; ECV_G = General factor explained common variance; ω_T = Coefficient omega total (total score reliability; ≥ 0.8 indicates high reliability); ω_S = Coefficient omega subscale (subscale reliability; > 0.8 indicates high reliability); ω_H = Coefficient omega hierarchical (general factor saturation; $\omega_H \geq 0.8$ or combination of $\omega_H \geq 0.7$ and $ECV_G \geq 0.60$ indicates strong general factor); ω_{HS} = Coefficient omega hierarchical subscale (specific factor saturation; ≥ 0.20/0.25 support the added value of a specific factor under conditions of high/low reliability, respectively); ECV_{SS} = Specific factor explained common variance for a subscale (≥ 0.30/0.45 support the added value of a specific factor under conditions of high/low reliability, respectively).

Supplemental Table S6

Meta-analytic effects and heterogeneity parameters for continuous correlates (Page 1 of 10)

**Correlations with SRS Total Score (Overall Autistic Traits)**										
**Sensory Construct**	**Correlate**	**n**	**r [95% CrI]**	**P_{ROPE}**	**log(BF_{ROPE})**	**Sig***	**τ^2 [95% CrI]**	**I^2 [95% CrI]**	**ICC [95% CrI]**	**95% PI for r**
**HYPER General**	**SRS**	**628**	**0.256 [0.073, 0.422]**	**0.050**	**1.289**	**SIG+**	**0.063 [3e-08, 0.203]**	**86.6% [59.4, 98.4]**	**0.157 [0.047, 0.325]**	**[-0.280, 0.689]**
**HYPER Auditory**	**SRS**	626	0.184 [0.032, 0.342]	0.131	0.242	INC	0.042 [6e-10, 0.138]	85.8% [50.5, 98.5]	0.065 [0.001, 0.174]	[-0.256, 0.603]
**HYPER Visual**	**SRS**	**481**	**0.254 [0.084, 0.398]**	**0.039**	**1.524**	**SIG+**	**0.019 [6e-12, 0.123]**	**70.9% [0.0, 94.1]**	**0.231 [0.074, 0.466]**	**[-0.142, 0.595]**
**HYPER Tactile**	**SRS**	**625**	**0.247 [0.064, 0.407]**	**0.055**	**1.182**	**SIG+**	**0.055 [2e-10, 0.190]**	**85.6% [39.5, 98.6]**	**0.169 [0.052, 0.335]**	**[-0.260, 0.686]**
HYPER Gustatory	SRS	482	0.216 [0.056, 0.375]	0.066	0.991	INC	0.022 [9e-13, 0.130]	73.9% [3.5, 97.5]	0.076 [0.003, 0.212]	[-0.163, 0.601]
HYPER Movement	SRS	356	0.205 [0.008, 0.396]	0.118	0.347	INC	0.027 [3e-12, 0.198]	70.2% [0.0, 94.6]	0.057 [0.000, 0.223]	[-0.251, 0.647]
**HYPO Speech**	**SRS**	**624**	**0.231 [0.104, 0.363]**	**0.024**	**2.063**	**SIG+**	**0.021 [3e-12, 0.096]**	**80.5% [10.2, 98.3]**	**0.063 [0.000, 0.171]**	**[-0.128, 0.559]**
HYPO Pain/Temp	SRS	480	0.169 [0.022, 0.318]	0.163	-0.016	INC	0.013 [1e-12, 0.093]	68.4% [0.0, 93.7]	0.179 [0.051, 0.390]	[-0.172, 0.503]
SEEK Visual	SRS	481	0.150 [-0.019, 0.312]	0.264	-0.632	INC	0.017 [2e-12, 0.115]	69.3% [0.0, 93.7]	0.101 [0.003, 0.267]	[-0.235, 0.519]
SEEK Tactile	SRS	614	0.172 [0.033, 0.295]	0.156	0.024	INC	0.007 [6e-12, 0.055]	57.6% [0.0, 91.6]	0.070 [0.010, 0.178]	[-0.115, 0.421]
SEEK Oral Tactile	SRS	385	0.128 [-0.090, 0.322]	0.352	-1.048	INC	0.039 [2e-11, 0.232]	77.6% [6.4, 98.6]	0.055 [0.000, 0.223]	[-0.403, 0.600]
SEEK Movement	SRS	622	0.161 [0.030, 0.295]	0.181	-0.165	INC	0.011 [6e-12, 0.065]	68.7% [0.0, 92.8]	0.045 [0.000, 0.132]	[-0.152, 0.446]
**Correlations with RBS-R RSM (Lower-order Repetitive Behaviors)**										
**Sensory Construct**	**Correlate**	**n**	**r [95% CrI]**	**P_{ROPE}**	**log(BF_{ROPE})**	**Sig***	**τ^2 [95% CrI]**	**I^2 [95% CrI]**	**ICC [95% CrI]**	**95% PI for r**
**HYPER General**	**RBS-R RSM**	**1587**	**0.251 [0.136, 0.378]**	**0.011**	**2.806**	**SIG++**	**0.005 [1e-13, 0.068]**	**54.4% [0.0, 94.0]**	**0.096 [0.018, 0.252]**	**[-0.010, 0.520]**
**HYPER Auditory**	**RBS-R RSM**	**1585**	**0.256 [0.069, 0.414]**	**0.049**	**1.293**	**SIG+**	**0.029 [1e-10, 0.156]**	**76.7% [5.4, 97.9]**	**0.144 [0.017, 0.371]**	**[-0.188, 0.640]**
**HYPER Visual**	**RBS-R RSM**	**1442**	**0.232 [0.074, 0.444]**	**0.043**	**1.433**	**SIG+**	**0.026 [6e-11, 0.203]**	**69.8% [9.1, 98.6]**	**0.117 [0.011, 0.335]**	**[-0.193, 0.669]**
**HYPER Tactile**	**RBS-R RSM**	**1565**	**0.245 [0.152, 0.348]**	**0.003**	**4.193**	**SIG++**	**0.004 [7e-13, 0.045]**	**55.3% [0.0, 94.0]**	**0.087 [0.012, 0.238]**	**[0.027, 0.463]**
HYPER Gustatory	RBS-R RSM	1423	0.146 [0.015, 0.334]	0.239	-0.503	INC	0.009 [3e-13, 0.109]	54.4% [0.0, 93.3]	0.132 [0.017, 0.367]	[-0.158, 0.516]
HYPER Olfactory	RBS-R RSM	1076	0.076 [-0.608, 0.647]	0.232	-0.470	INC	0.618 [3e-10, 15.344]	80.3% [2.5, 99.9]	0.834 [0.194, 0.995]	[-0.933, 0.997]
HYPER Movement	RBS-R RSM	1409	0.107 [-0.032, 0.221]	0.432	-1.378	ZERO+	0.003 [2e-13, 0.060]	40.1% [0.0, 93.3]	0.043 [0.000, 0.192]	[-0.162, 0.352]
**HYPO Speech**	**RBS-R RSM**	**1583**	**0.331 [0.191, 0.488]**	**0.002**	**4.516**	**SIG++**	**0.038 [0.002, 0.149]**	**81.5% [51.7, 97.8]**	**0.107 [0.016, 0.262]**	**[-0.081, 0.690]**
HYPO Pain/Temp	RBS-R RSM	1268	0.220 [-0.112, 0.405]	0.129	0.254	INC	0.017 [3e-13, 0.327]	48.4% [0.0, 94.7]	0.260 [0.016, 0.618]	[-0.416, 0.637]
**SEEK Visual**	**RBS-R RSM**	**1282**	**0.357 [-0.019, 0.555]**	**0.055**	**1.191**	**SIG+**	**0.024 [8e-11, 0.477]**	**47.2% [0.0, 94.7]**	**0.226 [0.007, 0.651]**	**[-0.322, 0.799]**
**SEEK Tactile**	**RBS-R RSM**	**1551**	**0.297 [0.217, 0.387]**	**0.001**	**5.580**	**SIG++**	**0.003 [6e-14, 0.033]**	**53.0% [0.0, 93.0]**	**0.012 [0.000, 0.069]**	**[0.125, 0.485]**
SEEK Oral Tactile	RBS-R RSM	1263	0.269 [-0.122, 0.553]	0.109	0.440	INC	0.079 [3e-11, 0.802]	70.0% [0.0, 96.0]	0.238 [0.000, 0.613]	[-0.507, 0.868]
**SEEK Movement**	**RBS-R RSM**	**1554**	**0.365 [0.248, 0.493]**	**0.000**	**6.126**	**SIG++**	**0.017 [2e-10, 0.079]**	**73.5% [26.0, 97.5]**	**0.039 [0.002, 0.133]**	**[0.079, 0.640]**

Table continues on following page.

Supplemental Table S6 (Continued: Page 2 of 10)

Correlations with RBS-R RSC (Higher-order Repetitive Behaviors)										
Sensory Construct	Correlate	*n*	*r* [95% CrI]	P_{ROPE}	log(BF_{ROPE})	Sig*	τ^2 [95% CrI]	I^2 [95% CrI]	ICC [95% CrI]	95% PI for *r*
HYPER General	RBS-R RSC	1587	0.358 [0.210, 0.484]	0.003	4.127	SIG++	0.021 [1e-11, 0.128]	76.0% [2.8, 97.8]	0.100 [0.015, 0.251]	[-0.017, 0.669]
HYPER Auditory	RBS-R RSC	1585	0.291 [0.094, 0.473]	0.031	1.773	SIG+	0.052 [1e-10, 0.260]	81.8% [22.2, 99.1]	0.125 [0.021, 0.313]	[-0.244, 0.733]
HYPER Visual	RBS-R RSC	1442	0.276 [0.142, 0.447]	0.010	2.971	SIG++	0.013 [6e-10, 0.136]	60.4% [0.0, 94.3]	0.077 [0.006, 0.265]	[-0.054, 0.620]
HYPER Tactile	RBS-R RSC	1565	0.321 [0.219, 0.433]	< 0.001	6.356	SIG++	0.005 [5e-11, 0.059]	56.0% [0.0, 93.8]	0.073 [0.009, 0.210]	[0.098, 0.560]
HYPER Gustatory	RBS-R RSC	1423	0.271 [0.129, 0.457]	0.014	2.568	SIG++	0.014 [2e-12, 0.187]	59.7% [0.0, 95.3]	0.113 [0.012, 0.336]	[-0.092, 0.664]
HYPER Olfactory	RBS-R RSC	1076	0.164 [-0.587, 0.677]	0.164	-0.037	INC	0.696 [3e-11, 16.585]	81.0% [2.6, 99.9]	0.851 [0.219, 0.996]	[-0.938, 0.997]
HYPER Movement	RBS-R RSC	1409	0.197 [0.060, 0.344]	0.057	1.151	SIG+	0.006 [3e-14, 0.086]	52.4% [0.0, 93.8]	0.039 [0.000, 0.179]	[-0.103, 0.499]
HYPO Speech	RBS-R RSC	1583	0.324 [0.165, 0.497]	0.006	3.420	SIG++	0.054 [0.003, 0.211]	84.1% [56.9, 98.3]	0.118 [0.017, 0.293]	[-0.156, 0.741]
HYPO Pain/Temp	RBS-R RSC	1268	0.158 [-0.308, 0.413]	0.226	-0.431	INC	0.057 [5e-12, 0.846]	61.0% [0.0, 95.9]	0.306 [0.048, 0.660]	[-0.680, 0.748]
SEEK Visual	RBS-R RSC	1282	0.249 [-0.029, 0.490]	0.070	0.933	INC	0.014 [2e-13, 0.379]	40.1% [0.0, 94.8]	0.187 [0.013, 0.617]	[-0.286, 0.744]
SEEK Tactile	RBS-R RSC	1551	0.205 [0.049, 0.383]	0.075	0.870	INC	0.029 [4e-11, 0.150]	77.3% [8.9, 98.2]	0.053 [0.000, 0.190]	[-0.215, 0.607]
SEEK Oral Tactile	RBS-R RSC	1263	0.128 [-0.375, 0.504]	0.272	-0.672	INC	0.245 [4e-08, 1.481]	80.6% [19.5, 99.3]	0.318 [0.044, 0.695]	[-0.766, 0.884]
SEEK Movement	RBS-R RSC	1554	0.257 [0.115, 0.422]	0.015	2.516	SIG++	0.025 [2e-09, 0.117]	76.1% [27.2, 97.9]	0.058 [0.007, 0.166]	[-0.103, 0.626]
Correlations with RBS-R SIB (Self-injurious Behaviors)										
Sensory Construct	Correlate	*n*	*r* [95% CrI]	P_{ROPE}	log(BF_{ROPE})	Sig*	τ^2 [95% CrI]	I^2 [95% CrI]	ICC [95% CrI]	95% PI for *r*
HYPER General	RBS-R SIB	1586	0.229 [0.138, 0.330]	0.006	3.499	SIG++	0.003 [2e-11, 0.038]	50.3% [0.0, 93.3]	0.087 [0.017, 0.222]	[0.032, 0.434]
HYPER Auditory	RBS-R SIB	1584	0.167 [0.054, 0.274]	0.100	0.520	INC	0.004 [7e-13, 0.049]	55.2% [0.0, 94.0]	0.117 [0.015, 0.311]	[-0.080, 0.401]
HYPER Visual	RBS-R SIB	1441	0.155 [0.038, 0.317]	0.131	0.216	INC	0.006 [1e-13, 0.103]	51.8% [0.0, 94.9]	0.080 [0.008, 0.256]	[-0.129, 0.495]
HYPER Tactile	RBS-R SIB	1564	0.247 [0.145, 0.363]	0.004	3.747	SIG++	0.004 [7e-16, 0.040]	49.6% [0.0, 91.5]	0.085 [0.012, 0.232]	[0.048, 0.476]
HYPER Gustatory	RBS-R SIB	1422	0.140 [-0.054, 0.375]	0.317	-0.905	INC	0.028 [8e-12, 0.279]	68.5% [4.4, 98.6]	0.163 [0.024, 0.412]	[-0.336, 0.661]
HYPER Olfactory	RBS-R SIB	1076	0.096 [-0.614, 0.642]	0.208	-0.327	INC	0.626 [7e-11, 15.518]	80.4% [2.5, 99.9]	0.837 [0.201, 0.996]	[-0.937, 0.995]
HYPER Movement	RBS-R SIB	1408	0.137 [-0.008, 0.312]	0.254	-0.595	INC	0.009 [3e-15, 0.114]	57.9% [0.0, 94.4]	0.054 [0.002, 0.215]	[-0.203, 0.496]
HYPO Speech	RBS-R SIB	1582	0.257 [0.117, 0.405]	0.014	2.616	SIG++	0.028 [4e-07, 0.114]	80.1% [48.6, 98.2]	0.086 [0.012, 0.230]	[-0.120, 0.615]
HYPO Pain/Temp	RBS-R SIB	1267	0.187 [-0.039, 0.384]	0.114	0.390	INC	0.009 [4e-11, 0.196]	42.9% [0.0, 94.4]	0.274 [0.027, 0.628]	[-0.247, 0.588]
SEEK Visual	RBS-R SIB	1281	0.184 [-0.088, 0.394]	0.159	0.010	INC	0.011 [2e-12, 0.280]	42.9% [0.0, 94.9]	0.196 [0.016, 0.584]	[-0.337, 0.623]
SEEK Tactile	RBS-R SIB	1550	0.191 [0.089, 0.327]	0.031	1.786	SIG+	0.005 [3e-13, 0.046]	54.7% [0.0, 91.5]	0.028 [0.000, 0.110]	[-0.025, 0.454]
SEEK Oral Tactile	RBS-R SIB	1262	0.203 [-0.079, 0.409]	0.133	0.222	INC	0.014 [2e-13, 0.284]	47.5% [0.0, 94.8]	0.224 [0.004, 0.621]	[-0.318, 0.649]
SEEK Movement	RBS-R SIB	1553	0.181 [0.073, 0.328]	0.057	1.152	SIG+	0.006 [8e-13, 0.046]	52.7% [0.0, 90.1]	0.043 [0.005, 0.137]	[-0.036, 0.449]

Table continues on following page.

Supplemental Table S6 (Continued: Page 3 of 10)

Correlations with Internalizing Symptoms

Sensory Construct	Correlate	*n*	*r* [95% CrI]	P_{ROPE}	log(BF_{ROPE})	Sig*	τ^2 [95% CrI]	I^2 [95% CrI]	ICC [95% CrI]	95% PI for *r*
**HYPER General**	**PSYC INT**	**765**	**0.218 [0.082, 0.347]**	**0.047**	**1.352**	**SIG+**	**0.030 [2e-10, 0.110]**	**85.2% [43.6, 98.5]**	**0.140 [0.041, 0.292]**	**[-0.181, 0.575]**
HYPER Auditory	PSYC INT	763	0.194 [0.069, 0.303]	0.067	0.985	INC	0.011 [4e-12, 0.069]	75.0% [3.2, 97.4]	0.037 [0.000, 0.121]	[-0.119, 0.475]
HYPER Visual	PSYC INT	623	0.190 [0.059, 0.303]	0.078	0.808	INC	0.015 [2e-13, 0.077]	78.5% [8.2, 98.2]	0.242 [0.084, 0.477]	[-0.141, 0.494]
HYPER Tactile	PSYC INT	765	0.171 [0.016, 0.314]	0.170	-0.080	INC	0.038 [1e-12, 0.134]	85.9% [42.3, 98.4]	0.168 [0.064, 0.318]	[-0.283, 0.566]
**HYPER Gustatory**	**PSYC INT**	**625**	**0.178 [0.086, 0.266]**	**0.045**	**1.396**	**SIG+**	**0.003 [1e-12, 0.029]**	**52.3% [0.0, 92.5]**	**0.071 [0.005, 0.203]**	**[-0.009, 0.364]**
HYPER Movement	PSYC INT	543	0.159 [0.020, 0.301]	0.177	-0.115	INC	0.017 [3e-11, 0.099]	74.9% [5.3, 98.2]	0.089 [0.006, 0.271]	[-0.202, 0.495]
**HYPO Speech**	**PSYC INT**	**761**	**0.286 [0.125, 0.453]**	**0.015**	**2.504**	**SIG++**	**0.080 [0.007, 0.232]**	**89.6% [72.4, 98.2]**	**0.148 [0.054, 0.285]**	**[-0.257, 0.743]**
HYPO Pain/Temp	PSYC INT	589	0.091 [-0.066, 0.244]	0.541	-1.828	ZERO+	0.022 [5e-13, 0.120]	78.1% [8.9, 98.7]	0.308 [0.123, 0.572]	[-0.308, 0.479]
SEEK Visual	PSYC INT	592	0.100 [-0.057, 0.240]	0.484	-1.607	ZERO+	0.015 [6e-12, 0.103]	71.9% [0.0, 94.7]	0.087 [0.001, 0.262]	[-0.280, 0.442]
SEEK Tactile	PSYC INT	755	0.129 [0.010, 0.263]	0.305	-0.837	INC	0.013 [1e-11, 0.080]	75.1% [3.4, 97.7]	0.080 [0.014, 0.194]	[-0.180, 0.461]
SEEK Oral Tactile	PSYC INT	532	0.105 [-0.093, 0.292]	0.451	-1.463	ZERO+	0.042 [2e-12, 0.178]	80.8% [36.3, 99.0]	0.130 [0.026, 0.347]	[-0.391, 0.558]
SEEK Movement	PSYC INT	761	0.134 [0.032, 0.235]	0.245	-0.519	INC	0.007 [2e-13, 0.046]	71.7% [0.0, 94.3]	0.084 [0.022, 0.190]	[-0.115, 0.386]

Correlations with Externalizing Symptoms

Sensory Construct	Correlate	*n*	*r* [95% CrI]	P_{ROPE}	log(BF_{ROPE})	Sig*	τ^2 [95% CrI]	I^2 [95% CrI]	ICC [95% CrI]	95% PI for *r*
**HYPER General**	**PSYC EXT**	**766**	**0.224 [0.099, 0.333]**	**0.032**	**1.727**	**SIG+**	**0.009 [8e-15, 0.052]**	**67.7% [0.0, 92.4]**	**0.114 [0.030, 0.250]**	**[-0.046, 0.480]**
**HYPER Auditory**	**PSYC EXT**	**764**	**0.190 [0.090, 0.289]**	**0.040**	**1.523**	**SIG+**	**0.005 [1e-11, 0.043]**	**64.8% [0.0, 93.8]**	**0.028 [0.000, 0.106]**	**[-0.046, 0.421]**
HYPER Visual	PSYC EXT	622	0.178 [0.055, 0.299]	0.096	0.558	INC	0.014 [3e-11, 0.066]	76.8% [10.0, 98.0]	0.242 [0.087, 0.478]	[-0.126, 0.477]
**HYPER Tactile**	**PSYC EXT**	**766**	**0.209 [0.088, 0.337]**	**0.036**	**1.624**	**SIG+**	**0.021 [4e-11, 0.089]**	**82.0% [11.2, 98.4]**	**0.144 [0.046, 0.291]**	**[-0.132, 0.544]**
**HYPER Gustatory**	**PSYC EXT**	**624**	**0.179 [0.073, 0.296]**	**0.058**	**1.121**	**SIG+**	**0.007 [2e-11, 0.056]**	**66.9% [0.0, 94.1]**	**0.079 [0.008, 0.214]**	**[-0.073, 0.451]**
HYPER Movement	PSYC EXT	543	0.081 [-0.075, 0.224]	0.599	-2.073	ZERO+	0.016 [3e-11, 0.100]	73.7% [3.9, 97.9]	0.088 [0.007, 0.254]	[-0.290, 0.430]
**HYPO Speech**	**PSYC EXT**	**762**	**0.276 [0.144, 0.411]**	**0.008**	**3.200**	**SIG++**	**0.041 [1e-07, 0.130]**	**87.5% [58.6, 98.3]**	**0.114 [0.036, 0.229]**	**[-0.152, 0.642]**
HYPO Pain/Temp	PSYC EXT	588	0.121 [-0.030, 0.284]	0.379	-1.176	ZERO+	0.024 [7e-13, 0.114]	78.5% [25.1, 98.7]	0.300 [0.116, 0.558]	[-0.271, 0.507]
SEEK Visual	PSYC EXT	591	0.079 [-0.058, 0.213]	0.622	-2.166	ZERO+	0.008 [1e-12, 0.067]	63.1% [0.0, 93.3]	0.078 [0.000, 0.240]	[-0.232, 0.369]
**SEEK Tactile**	**PSYC EXT**	**756**	**0.206 [0.073, 0.344]**	**0.050**	**1.291**	**SIG+**	**0.029 [8e-16, 0.121]**	**84.4% [25.4, 99.0]**	**0.093 [0.017, 0.218]**	**[-0.184, 0.584]**
SEEK Oral Tactile	PSYC EXT	532	0.138 [-0.053, 0.317]	0.315	-0.882	INC	0.037 [5e-10, 0.168]	80.1% [31.8, 98.7]	0.138 [0.029, 0.356]	[-0.340, 0.570]
**SEEK Movement**	**PSYC EXT**	**762**	**0.242 [0.119, 0.368]**	**0.013**	**2.644**	**SIG++**	**0.025 [2e-10, 0.099]**	**83.6% [17.7, 98.6]**	**0.113 [0.032, 0.234]**	**[-0.117, 0.583]**

Table continues on following page.

Supplemental Table S6 (Continued: Page 4 of 10)

Sensory Construct	Correlate	*n*	*r* [95% CrI]	P_{ROPE}	log(BF_{ROPE})	Sig*	τ^2 [95% CrI]	I^2 [95% CrI]	ICC [95% CrI]	95% PI for *r*
**Correlations with ADHD Features**										
HYPER General	PSYC ADHD	717	0.177 [0.072, 0.273]	0.071	0.892	INC	0.006 [1e-13, 0.041]	68.3% [0.0, 93.5]	0.131 [0.036, 0.291]	[-0.065, 0.403]
HYPER Auditory	PSYC ADHD	715	0.179 [0.061, 0.275]	0.086	0.718	INC	0.004 [3e-12, 0.039]	58.2% [0.0, 92.4]	0.078 [0.002, 0.224]	[-0.059, 0.392]
HYPER Visual	PSYC ADHD	574	0.133 [0.031, 0.231]	0.250	-0.555	INC	0.003 [4e-11, 0.027]	47.6% [0.0, 90.7]	0.195 [0.064, 0.405]	[-0.060, 0.321]
HYPER Tactile	PSYC ADHD	717	0.152 [0.050, 0.247]	0.146	0.106	INC	0.005 [6e-14, 0.036]	64.9% [0.0, 93.0]	0.151 [0.046, 0.317]	[-0.073, 0.370]
HYPER Gustatory	PSYC ADHD	576	0.127 [0.030, 0.229]	0.283	-0.748	INC	0.003 [6e-13, 0.028]	51.1% [0.0, 91.2]	0.069 [0.000, 0.209]	[-0.070, 0.320]
HYPER Movement	PSYC ADHD	494	0.110 [-0.009, 0.222]	0.428	-1.360	ZERO+	0.003 [3e-14, 0.037]	47.7% [0.0, 91.1]	0.049 [0.000, 0.168]	[-0.120, 0.325]
**HYPO Speech**	**PSYC ADHD**	**713**	**0.297 [0.135, 0.449]**	**0.013**	**2.696**	**SIG++**	**0.069 [0.009, 0.193]**	**89.1% [73.5, 97.9]**	**0.192 [0.080, 0.343]**	**[-0.226, 0.719]**
HYPO Pain/Temp	PSYC ADHD	496	0.073 [-0.106, 0.252]	0.605	-2.097	ZERO+	0.033 [2e-12, 0.159]	80.1% [26.1, 99.0]	0.341 [0.140, 0.600]	[-0.378, 0.519]
SEEK Visual	PSYC ADHD	499	0.124 [-0.072, 0.306]	0.384	-1.191	ZERO+	0.033 [6e-11, 0.141]	76.8% [25.3, 98.3]	0.110 [0.015, 0.286]	[-0.326, 0.543]
**SEEK Tactile**	**PSYC ADHD**	**706**	**0.218 [0.077, 0.360]**	**0.043**	**1.409**	**SIG+**	**0.031 [5e-10, 0.133]**	**83.7% [20.1, 98.9]**	**0.108 [0.014, 0.248]**	**[-0.197, 0.599]**
**SEEK Oral Tactile**	**PSYC ADHD**	**440**	**0.285 [0.156, 0.402]**	**0.009**	**3.110**	**SIG++**	**0.007 [2e-13, 0.081]**	**58.5% [0.0, 94.1]**	**0.161 [0.020, 0.428]**	**[-0.005, 0.552]**
**SEEK Movement**	**PSYC ADHD**	**713**	**0.229 [0.078, 0.370]**	**0.044**	**1.416**	**SIG+**	**0.043 [3e-09, 0.138]**	**86.9% [61.1, 98.3]**	**0.108 [0.027, 0.238]**	**[-0.212, 0.631]**
**Correlations with Total Psychiatric Symptoms**										
**Sensory Construct**	**Correlate**	***n***	***r* [95% CrI]**	P_{ROPE}	**log(BF_{ROPE})**	**Sig***	τ^2 **[95% CrI]**	I^2 **[95% CrI]**	**ICC [95% CrI]**	**95% PI for *r***
**HYPER General**	**PSYC Total**	**671**	**0.245 [0.097, 0.371]**	**0.033**	**1.708**	**SIG+**	**0.028 [1e-10, 0.101]**	**82.7% [31.3, 98.2]**	**0.153 [0.042, 0.336]**	**[-0.147, 0.584]**
**HYPER Auditory**	**PSYC Total**	**670**	**0.233 [0.099, 0.357]**	**0.030**	**1.792**	**SIG+**	**0.019 [6e-14, 0.106]**	**79.3% [4.3, 98.2]**	**0.075 [0.000, 0.208]**	**[-0.141, 0.561]**
HYPER Visual	PSYC Total	621	0.189 [0.046, 0.312]	0.096	0.597	INC	0.016 [8e-12, 0.090]	76.3% [6.1, 98.1]	0.240 [0.085, 0.465]	[-0.160, 0.509]
HYPER Tactile	PSYC Total	671	0.196 [0.042, 0.336]	0.098	0.575	INC	0.040 [1e-10, 0.131]	86.6% [51.2, 98.3]	0.185 [0.064, 0.358]	[-0.253, 0.593]
**HYPER Gustatory**	**PSYC Total**	**622**	**0.203 [0.093, 0.327]**	**0.031**	**1.770**	**SIG+**	**0.010 [4e-13, 0.065]**	**71.7% [0.0, 94.0]**	**0.118 [0.019, 0.296]**	**[-0.074, 0.494]**
HYPER Movement	PSYC Total	543	0.178 [0.050, 0.303]	0.101	0.529	INC	0.009 [3e-12, 0.069]	67.4% [0.0, 93.9]	0.069 [0.004, 0.214]	[-0.119, 0.464]
**HYPO Speech**	**PSYC Total**	**669**	**0.329 [0.197, 0.460]**	**0.003**	**4.240**	**SIG++**	**0.039 [3e-10, 0.146]**	**86.1% [20.9, 98.8]**	**0.122 [0.029, 0.259]**	**[-0.083, 0.697]**
HYPO Pain/Temp	PSYC Total	587	0.156 [-0.019, 0.325]	0.211	-0.353	INC	0.027 [5e-13, 0.186]	77.8% [5.5, 98.8]	0.337 [0.138, 0.594]	[-0.307, 0.585]
SEEK Visual	PSYC Total	590	0.133 [-0.032, 0.274]	0.316	-0.892	INC	0.015 [1e-14, 0.105]	70.8% [0.0, 94.3]	0.085 [0.000, 0.247]	[-0.262, 0.467]
SEEK Tactile	PSYC Total	665	0.194 [0.052, 0.332]	0.081	0.761	INC	0.025 [1e-10, 0.119]	81.9% [13.6, 98.8]	0.092 [0.016, 0.225]	[-0.203, 0.560]
**SEEK Oral Tactile**	**PSYC Total**	**532**	**0.259 [0.140, 0.371]**	**0.013**	**2.717**	**SIG++**	**0.004 [2e-14, 0.055]**	**46.7% [0.0, 92.8]**	**0.188 [0.029, 0.473]**	**[0.004, 0.477]**
**SEEK Movement**	**PSYC Total**	**667**	**0.251 [0.119, 0.379]**	**0.018**	**2.331**	**SIG++**	**0.025 [9e-13, 0.106]**	**83.0% [14.5, 98.6]**	**0.136 [0.037, 0.292]**	**[-0.130, 0.589]**

Table continues on following page.

Supplemental Table S6 (Continued: Page 5 of 10)

Correlations with Chronological Age

Sensory Construct	Correlate	n	r [95% CrI]	P_{ROPE}	$\log(BF_{ROPE})$	Sig*	τ^2 [95% CrI]	I^2 [95% CrI]	ICC [95% CrI]	95% PI for r
HYPER General	Age	3866	0.009 [-0.061, 0.072]	0.994	-6.830	ZERO++	0.006 [2e-09, 0.021]	83.8% [48.4, 98.1]	0.082 [0.036, 0.152]	[-0.185, 0.196]
HYPER Auditory	Age	3860	-0.037 [-0.127, 0.049]	0.925	-4.165	ZERO++	0.017 [2e-10, 0.059]	89.5% [62.3, 98.8]	0.059 [0.016, 0.134]	[-0.343, 0.263]
HYPER Visual	Age	3700	0.013 [-0.034, 0.059]	0.999	-9.277	ZERO++	4e-04 [3e-15, 0.005]	44.1% [0.0, 90.4]	0.092 [0.039, 0.170]	[-0.078, 0.097]
HYPER Tactile	Age	3837	0.014 [-0.057, 0.073]	0.995	-6.995	ZERO++	0.004 [2e-11, 0.019]	76.5% [6.2, 97.7]	0.092 [0.038, 0.170]	[-0.169, 0.173]
HYPER Gustatory	Age	3682	-0.065 [-0.123, -0.008]	0.894	-3.806	ZERO++	0.002 [2e-12, 0.014]	73.9% [3.8, 97.3]	0.044 [0.013, 0.097]	[-0.211, 0.080]
HYPER Olfactory	Age	2467	0.205 [-0.128, 0.498]	0.165	-0.047	INC	0.050 [4e-04, 0.567]	64.1% [20.6, 99.5]	0.214 [0.016, 0.671]	[-0.453, 0.785]
HYPER Movement	Age	3524	0.038 [-0.036, 0.110]	0.960	-4.833	ZERO++	0.006 [4e-11, 0.024]	81.9% [32.9, 98.4]	0.036 [0.010, 0.079]	[-0.160, 0.238]
HYPO Speech	Age	3855	-0.129 [-0.189, -0.076]	0.141	0.151	INC	0.003 [4e-12, 0.014]	76.5% [12.4, 97.9]	0.058 [0.024, 0.109]	[-0.278, 0.013]
HYPO Pain/Temp	Age	3484	-0.020 [-0.133, 0.078]	0.915	-4.036	ZERO++	0.018 [0.002, 0.056]	86.4% [68.6, 97.7]	0.157 [0.066, 0.286]	[-0.333, 0.286]
SEEK Visual	Age	3502	-0.114 [-0.205, -0.011]	0.385	-1.201	ZERO+	0.014 [0.001, 0.046]	84.9% [62.9, 97.4]	0.062 [0.020, 0.134]	[-0.382, 0.172]
SEEK Tactile	Age	3816	-0.123 [-0.188, -0.060]	0.221	-0.406	INC	0.004 [6e-11, 0.018]	78.5% [15.0, 98.0]	0.035 [0.008, 0.075]	[-0.291, 0.042]
**SEEK Oral Tactile**	**Age**	**3367**	**-0.164 [-0.248, -0.091]**	**0.034**	**1.677**	**SIG+**	**0.006 [6e-11, 0.025]**	**77.0% [19.0, 97.9]**	**0.035 [0.006, 0.087]**	**[-0.373, 0.017]**
**SEEK Movement**	**Age**	**3824**	**-0.187 [-0.274, -0.100]**	**0.026**	**1.963**	**SIG+**	**0.022 [0.005, 0.056]**	**91.1% [80.2, 97.9]**	**0.079 [0.035, 0.147]**	**[-0.479, 0.129]**

Correlations with Verbal Cognitive Scores (IQ or DQ)

Sensory Construct	Correlate	n	r [95% CrI]	P_{ROPE}	$\log(BF_{ROPE})$	Sig*	τ^2 [95% CrI]	I^2 [95% CrI]	ICC [95% CrI]	95% PI for r
HYPER General	VIQ/DQ	1038	0.036 [-0.056, 0.120]	0.931	-4.269	ZERO++	0.005 [5e-13, 0.036]	70.9% [0.0, 94.8]	0.049 [0.005, 0.117]	[-0.197, 0.255]
HYPER Auditory	VIQ/DQ	1037	0.073 [-0.025, 0.162]	0.729	-2.674	ZERO++	0.004 [4e-13, 0.032]	65.8% [0.0, 93.3]	0.033 [0.000, 0.095]	[-0.146, 0.281]
HYPER Visual	VIQ/DQ	907	0.085 [-0.014, 0.174]	0.632	-2.197	ZERO+	0.003 [6e-14, 0.030]	51.8% [0.0, 92.8]	0.077 [0.020, 0.170]	[-0.126, 0.271]
HYPER Tactile	VIQ/DQ	1035	0.019 [-0.064, 0.104]	0.965	-4.961	ZERO++	0.003 [1e-12, 0.030]	64.9% [0.0, 94.2]	0.033 [0.000, 0.096]	[-0.182, 0.224]
HYPER Gustatory	VIQ/DQ	905	0.002 [-0.099, 0.114]	0.934	-4.287	ZERO++	0.006 [2e-12, 0.050]	68.3% [0.0, 94.4]	0.044 [0.002, 0.120]	[-0.242, 0.282]
HYPER Olfactory	VIQ/DQ	100	0.004 [-0.414, 0.408]	0.423	-1.333	ZERO+	0.053 [9e-14, 1.090]	52.6% [0.0, 95.8]	0.384 [0.020, 0.854]	[-0.760, 0.731]
HYPER Movement	VIQ/DQ	853	0.033 [-0.068, 0.133]	0.902	-3.893	ZERO++	0.003 [5e-15, 0.032]	53.8% [0.0, 92.4]	0.045 [0.002, 0.123]	[-0.180, 0.239]
HYPO Speech	VIQ/DQ	1037	0.055 [-0.046, 0.166]	0.803	-3.067	ZERO++	0.014 [5e-13, 0.071]	83.1% [7.0, 98.5]	0.078 [0.025, 0.160]	[-0.250, 0.387]
HYPO Pain/Temp	VIQ/DQ	803	-0.101 [-0.236, 0.031]	0.492	-1.626	ZERO+	0.026 [0.002, 0.080]	84.4% [62.3, 97.1]	0.140 [0.055, 0.269]	[-0.461, 0.258]
SEEK Visual	VIQ/DQ	803	-0.096 [-0.211, 0.020]	0.525	-1.776	ZERO+	0.008 [8e-13, 0.048]	68.6% [0.0, 93.1]	0.021 [0.000, 0.086]	[-0.356, 0.170]
SEEK Tactile	VIQ/DQ	1016	-0.089 [-0.185, 0.007]	0.595	-2.038	ZERO+	0.007 [3e-11, 0.041]	74.6% [0.0, 94.4]	0.042 [0.003, 0.102]	[-0.332, 0.154]
SEEK Oral Tactile	VIQ/DQ	760	-0.132 [-0.248, -0.008]	0.291	-0.768	INC	0.008 [3e-12, 0.054]	67.0% [0.0, 93.3]	0.058 [0.007, 0.157]	[-0.391, 0.150]
SEEK Movement	VIQ/DQ	1033	-0.043 [-0.144, 0.060]	0.868	-3.525	ZERO++	0.011 [4e-12, 0.047]	79.6% [12.6, 97.9]	0.070 [0.023, 0.143]	[-0.306, 0.233]

Table continues on following page.

Supplemental Table S6 (Continued: Page 6 of 10)

Correlations with Verbal Cognitive Scores (IQ Only)

Sensory Construct	Correlate	n	r [95% CrI]	P_{ROPE}	log(BF_{ROPE})	Sig*	τ^2 [95% CrI]	I^2 [95% CrI]	ICC [95% CrI]	95% PI for r
HYPER General	VIQ	808	0.032 [-0.061, 0.122]	0.928	-4.215	ZERO++	0.002 [3e-13, 0.025]	52.9% [0.0, 91.9]	0.029 [0.000, 0.095]	[-0.157, 0.218]
HYPER Auditory	VIQ	808	-0.015 [-0.108, 0.076]	0.955	-4.704	ZERO++	0.003 [3e-14, 0.030]	60.3% [0.0, 93.0]	0.028 [0.000, 0.094]	[-0.222, 0.184]
HYPER Visual	VIQ	737	0.054 [-0.052, 0.156]	0.823	-3.206	ZERO++	0.005 [5e-14, 0.047]	66.0% [0.0, 94.3]	0.074 [0.013, 0.178]	[-0.202, 0.298]
HYPER Tactile	VIQ	805	0.045 [-0.045, 0.134]	0.887	-3.716	ZERO++	0.003 [3e-15, 0.028]	58.2% [0.0, 92.9]	0.023 [0.000, 0.087]	[-0.145, 0.246]
HYPER Gustatory	VIQ	735	-0.005 [-0.116, 0.112]	0.917	-4.076	ZERO++	0.009 [9e-14, 0.063]	73.1% [0.0, 94.9]	0.041 [0.000, 0.121]	[-0.293, 0.291]
HYPER Olfactory	VIQ	683	0.017 [-0.109, 0.133]	0.889	-3.744	ZERO++	0.008 [5e-12, 0.050]	67.9% [0.0, 92.9]	0.031 [0.000, 0.106]	[-0.262, 0.282]
HYPER Movement	VIQ	808	0.121 [0.031, 0.216]	0.314	-0.893	INC	0.005 [5e-12, 0.038]	69.2% [0.0, 94.1]	0.090 [0.028, 0.189]	[-0.096, 0.352]
HYPO Speech	VIQ	645	-0.050 [-0.162, 0.056]	0.816	-3.146	ZERO++	0.007 [4e-12, 0.037]	68.3% [0.0, 92.1]	0.165 [0.062, 0.327]	[-0.294, 0.184]
HYPO Pain/Temp	VIQ	645	-0.073 [-0.180, 0.042]	0.688	-2.446	ZERO++	0.003 [6e-14, 0.028]	44.2% [0.0, 89.5]	0.018 [0.000, 0.078]	[-0.278, 0.127]
SEEK Visual	VIQ	791	-0.030 [-0.133, 0.077]	0.904	-3.916	ZERO++	0.005 [2e-12, 0.042]	64.8% [0.0, 93.5]	0.049 [0.004, 0.126]	[-0.258, 0.228]
SEEK Tactile	VIQ	603	-0.054 [-0.163, 0.059]	0.795	-3.008	ZERO++	0.003 [9e-13, 0.033]	47.5% [0.0, 91.0]	0.075 [0.010, 0.203]	[-0.269, 0.155]
SEEK Oral Tactile	VIQ	804	0.029 [-0.068, 0.121]	0.931	-4.249	ZERO++	0.003 [8e-18, 0.023]	51.6% [0.0, 90.9]	0.055 [0.010, 0.134]	[-0.162, 0.209]
SEEK Movement	VIQ	808	0.032 [-0.061, 0.122]	0.928	-4.215	ZERO++	0.002 [3e-13, 0.025]	52.9% [0.0, 91.9]	0.029 [0.000, 0.095]	[-0.157, 0.218]

Correlations with Nonverbal Cognitive Scores (IQ or DQ)

Sensory Construct	Correlate	n	r [95% CrI]	P_{ROPE}	log(BF_{ROPE})	Sig*	τ^2 [95% CrI]	I^2 [95% CrI]	ICC [95% CrI]	95% PI for r
HYPER General	NVIQ/DQ	1193	0.044 [-0.037, 0.123]	0.923	-4.160	ZERO++	0.003 [5e-14, 0.024]	64.8% [0.0, 93.5]	0.044 [0.006, 0.106]	[-0.145, 0.229]
HYPER Auditory	NVIQ/DQ	1192	0.032 [-0.054, 0.111]	0.954	-4.700	ZERO++	0.002 [2e-14, 0.020]	53.7% [0.0, 91.9]	0.027 [0.000, 0.083]	[-0.142, 0.197]
HYPER Visual	NVIQ/DQ	1063	0.093 [0.007, 0.178]	0.568	-1.929	ZERO+	0.003 [8e-14, 0.030]	57.0% [0.0, 94.0]	0.072 [0.020, 0.154]	[-0.102, 0.291]
HYPER Tactile	NVIQ/DQ	1190	0.042 [-0.037, 0.123]	0.924	-4.146	ZERO++	0.003 [3e-12, 0.033]	66.3% [0.0, 95.2]	0.034 [0.000, 0.092]	[-0.162, 0.249]
HYPER Gustatory	NVIQ/DQ	1061	0.020 [-0.072, 0.113]	0.946	-4.532	ZERO++	0.003 [6e-12, 0.029]	56.9% [0.0, 93.0]	0.031 [0.000, 0.086]	[-0.160, 0.234]
HYPER Olfactory	NVIQ/DQ	133	0.012 [-0.346, 0.387]	0.478	-1.582	ZERO+	0.041 [3e-12, 0.850]	52.7% [0.0, 95.8]	0.346 [0.016, 0.820]	[-0.667, 0.735]
HYPER Movement	NVIQ/DQ	1009	0.012 [-0.084, 0.134]	0.915	-4.044	ZERO++	0.006 [2e-11, 0.051]	64.1% [0.0, 94.2]	0.051 [0.007, 0.137]	[-0.227, 0.292]
HYPO Speech	NVIQ/DQ	1191	0.092 [0.010, 0.184]	0.574	-1.975	ZERO+	0.005 [1e-11, 0.032]	70.2% [0.0, 93.9]	0.061 [0.019, 0.127]	[-0.104, 0.318]
HYPO Pain/Temp	NVIQ/DQ	958	-0.098 [-0.214, 0.021]	0.515	-1.741	ZERO+	0.020 [0.003, 0.061]	84.7% [64.5, 96.9]	0.134 [0.053, 0.255]	[-0.407, 0.232]
SEEK Visual	NVIQ/DQ	958	-0.074 [-0.185, 0.040]	0.679	-2.415	ZERO++	0.011 [1e-09, 0.052]	76.6% [9.0, 97.6]	0.032 [0.000, 0.103]	[-0.349, 0.212]
SEEK Tactile	NVIQ/DQ	1171	-0.087 [-0.182, -0.004]	0.613	-2.113	ZERO+	0.006 [1e-11, 0.037]	72.4% [0.0, 94.6]	0.042 [0.005, 0.101]	[-0.322, 0.129]
SEEK Oral Tactile	NVIQ/DQ	916	-0.096 [-0.195, 0.018]	0.530	-1.777	ZERO+	0.005 [1e-13, 0.037]	62.3% [0.0, 92.5]	0.055 [0.007, 0.147]	[-0.318, 0.142]
SEEK Movement	NVIQ/DQ	1188	-0.026 [-0.118, 0.067]	0.940	-4.408	ZERO++	0.008 [3e-12, 0.040]	78.2% [8.8, 98.0]	0.061 [0.020, 0.127]	[-0.269, 0.224]

Table continues on following page.

Supplemental Table S6 (Continued: Page 7 of 10)

Correlations with Nonverbal Cognitive Scores (IQ Only)

Sensory Construct	Correlate	*n*	*r* [95% CrI]	P_{ROPE}	log(BF_{ROPE})	Sig*	τ^2 [95% CrI]	I^2 [95% CrI]	ICC [95% CrI]	95% PI for *r*
HYPER General	NVIQ	967	0.028 [-0.050, 0.106]	0.966	-4.999	ZERO++	0.001 [3e-15, 0.013]	45.7% [0.0, 89.0]	0.026 [0.000, 0.079]	[-0.117, 0.167]
HYPER Auditory	NVIQ	967	-0.058 [-0.136, 0.020]	0.855	-3.445	ZERO++	0.001 [2e-12, 0.011]	42.0% [0.0, 87.5]	0.02 [0.00, 0.07]	[-0.192, 0.076]
HYPER Visual	NVIQ	897	0.062 [-0.023, 0.143]	0.825	-3.215	ZERO++	0.003 [2e-13, 0.029]	63.6% [0.0, 94.0]	0.057 [0.010, 0.136]	[-0.130, 0.262]
HYPER Tactile	NVIQ	964	0.045 [-0.030, 0.124]	0.917	-4.076	ZERO++	0.002 [1e-12, 0.018]	55.9% [0.0, 91.9]	0.025 [0.000, 0.077]	[-0.108, 0.210]
HYPER Gustatory	NVIQ	895	0.022 [-0.060, 0.104]	0.964	-4.958	ZERO++	0.002 [3e-12, 0.021]	56.6% [0.0, 92.3]	0.022 [0.000, 0.070]	[-0.144, 0.198]
HYPER Olfactory	NVIQ	108	0.006 [-0.327, 0.342]	0.509	-1.708	ZERO+	0.029 [2e-12, 0.588]	48.8% [0.0, 95.0]	0.260 [0.000, 0.772]	[-0.623, 0.655]
HYPER Movement	NVIQ	843	0.001 [-0.091, 0.098]	0.961	-4.854	ZERO++	0.003 [7e-13, 0.028]	55.5% [0.0, 92.4]	0.031 [0.000, 0.097]	[-0.186, 0.211]
HYPO Speech	NVIQ	966	0.144 [0.069, 0.216]	0.12	0.331	INC	0.002 [1e-12, 0.015]	54.1% [0.0, 90.8]	0.074 [0.024, 0.152]	[0.002, 0.292]
HYPO Pain/Temp	NVIQ	804	-0.069 [-0.180, 0.040]	0.713	-2.584	ZERO++	0.015 [9e-10, 0.051]	82.9% [36.8, 98.0]	0.151 [0.060, 0.282]	[-0.367, 0.223]
SEEK Visual	NVIQ	804	-0.059 [-0.154, 0.034]	0.809	-3.096	ZERO++	0.003 [1e-11, 0.028]	59.6% [0.0, 92.5]	0.021 [0.000, 0.077]	[-0.262, 0.142]
SEEK Tactile	NVIQ	950	-0.045 [-0.128, 0.039]	0.906	-3.928	ZERO++	0.003 [3e-12, 0.024]	62.6% [0.0, 93.1]	0.044 [0.005, 0.108]	[-0.229, 0.144]
SEEK Oral Tactile	NVIQ	763	-0.038 [-0.130, 0.064]	0.909	-3.962	ZERO++	0.002 [8e-13, 0.020]	43.1% [0.0, 88.9]	0.064 [0.008, 0.167]	[-0.207, 0.137]
SEEK Movement	NVIQ	963	0.016 [-0.065, 0.096]	0.977	-5.434	ZERO++	0.002 [3e-11, 0.018]	54.1% [0.0, 91.6]	0.052 [0.010, 0.121]	[-0.150, 0.175]

Correlations with Full-scale Cognitive Scores (IQ or DQ)

Sensory Construct	Correlate	*n*	*r* [95% CrI]	P_{ROPE}	log(BF_{ROPE})	Sig*	τ^2 [95% CrI]	I^2 [95% CrI]	ICC [95% CrI]	95% PI for *r*
HYPER General	FSIQ/DQ	1028	0.034 [-0.053, 0.118]	0.943	-4.460	ZERO++	0.002 [3e-12, 0.022]	53.7% [0.0, 92.1]	0.044 [0.005, 0.109]	[-0.147, 0.207]
HYPER Auditory	FSIQ/DQ	1027	0.008 [-0.096, 0.110]	0.941	-4.457	ZERO++	0.005 [6e-13, 0.039]	66.0% [0.0, 93.3]	0.037 [0.000, 0.102]	[-0.236, 0.239]
HYPER Visual	FSIQ/DQ	952	0.065 [-0.035, 0.156]	0.781	-2.926	ZERO++	0.002 [1e-12, 0.028]	50.3% [0.0, 92.2]	0.076 [0.021, 0.165]	[-0.144, 0.246]
HYPER Tactile	FSIQ/DQ	1024	0.034 [-0.045, 0.115]	0.946	-4.508	ZERO++	0.002 [9e-14, 0.017]	50.0% [0.0, 91.1]	0.030 [0.000, 0.089]	[-0.120, 0.192]
HYPER Gustatory	FSIQ/DQ	949	-0.014 [-0.095, 0.074]	0.975	-5.303	ZERO++	0.002 [3e-12, 0.019]	51.0% [0.0, 91.1]	0.038 [0.001, 0.102]	[-0.172, 0.161]
HYPER Olfactory	FSIQ/DQ	113	0.028 [-0.422, 0.454]	0.392	-1.220	ZERO+	0.085 [1e-12, 1.435]	60.5% [0.0, 96.3]	0.343 [0.000, 0.822]	[-0.769, 0.807]
HYPER Movement	FSIQ/DQ	880	-0.014 [-0.105, 0.082]	0.956	-4.724	ZERO++	0.002 [1e-14, 0.024]	48.9% [0.0, 91.2]	0.037 [0.002, 0.099]	[-0.197, 0.171]
HYPO Speech	FSIQ/DQ	1026	0.097 [-0.001, 0.203]	0.521	-1.742	ZERO+	0.010 [3e-13, 0.054]	78.4% [5.6, 97.9]	0.078 [0.021, 0.167]	[-0.164, 0.387]
HYPO Pain/Temp	FSIQ/DQ	843	-0.076 [-0.199, 0.048]	0.650	-2.281	ZERO+	0.019 [9e-04, 0.062]	82.8% [56.7, 97.2]	0.133 [0.051, 0.259]	[-0.397, 0.247]
SEEK Visual	FSIQ/DQ	844	-1e-01 [-2e-01, 5e-04]	0.403	-1.267	ZERO+	0.008 [4e-15, 0.050]	70.1% [0.0, 93.5]	0.033 [0.000, 0.117]	[-0.375, 0.154]
SEEK Tactile	FSIQ/DQ	1011	-0.071 [-0.170, 0.023]	0.729	-2.665	ZERO++	0.007 [9e-13, 0.041]	72.9% [0.0, 94.3]	0.046 [0.006, 0.110]	[-0.317, 0.170]
SEEK Oral Tactile	FSIQ/DQ	784	-0.150 [-0.255, -0.035]	0.184	-0.169	INC	0.004 [4e-13, 0.037]	51.1% [0.0, 91.6]	0.047 [0.004, 0.132]	[-0.366, 0.078]
SEEK Movement	FSIQ/DQ	1022	-0.021 [-0.137, 0.092]	0.896	-3.827	ZERO++	0.016 [1e-11, 0.066]	82.8% [19.8, 98.2]	0.083 [0.025, 0.168]	[-0.339, 0.298]

Table continues on following page.

Supplemental Table S6 (Continued: Page 8 of 10)

Correlations with Full-scale Cognitive Scores (IQ Only)										
**Sensory Construct**	**Correlate**	***n***	***r* [95% CrI]**	***P*ROPE**	**log(*BF*ROPE)**	**Sig***	**τ² [95% CrI]**	***I*² [95% CrI]**	**ICC [95% CrI]**	**95% PI for *r***
HYPER General	FSIQ	852	0.011 [-0.074, 0.096]	0.975	-5.311	ZERO++	0.002 [5e-12, 0.018]	47.3% [0.0, 90.5]	0.032 [0.000, 0.094]	[-0.148, 0.178]
HYPER Auditory	FSIQ	851	-0.054 [-0.151, 0.036]	0.831	-3.25	ZERO++	0.003 [1e-13, 0.026]	58.0% [0.0, 92.0]	0.030 [0.000, 0.093]	[-0.253, 0.138]
HYPER Visual	FSIQ	782	0.041 [-0.059, 0.131]	0.901	-3.859	ZERO++	0.003 [3e-13, 0.030]	58.1% [0.0, 92.6]	0.069 [0.012, 0.159]	[-0.175, 0.233]
HYPER Tactile	FSIQ	848	0.026 [-0.057, 0.111]	0.956	-4.75	ZERO++	0.002 [5e-13, 0.018]	48.0% [0.0, 90.8]	0.025 [0.000, 0.096]	[-0.132, 0.187]
HYPER Gustatory	FSIQ	779	-0.001 [-0.092, 0.091]	0.967	-5.05	ZERO++	0.003 [3e-14, 0.030]	61.7% [0.0, 93.2]	0.034 [0.000, 0.100]	[-0.201, 0.207]
HYPER Olfactory	FSIQ	710	-0.002 [-0.114, 0.106]	0.925	-4.179	ZERO++	0.003 [3e-13, 0.028]	48.1% [0.0, 89.8]	0.025 [0.000, 0.086]	[-0.209, 0.201]
HYPER Movement	FSIQ	850	0.141 [0.056, 0.226]	0.161	0.004	INC	0.003 [4e-13, 0.031]	63.4% [0.0, 94.1]	0.093 [0.029, 0.192]	[-0.056, 0.346]
HYPO Speech	FSIQ	678	-0.043 [-0.147, 0.063]	0.857	-3.472	ZERO++	0.005 [4e-15, 0.032]	64.0% [0.0, 91.8]	0.157 [0.059, 0.307]	[-0.264, 0.179]
HYPO Pain/Temp	FSIQ	679	-0.067 [-0.186, 0.040]	0.716	-2.582	ZERO++	0.003 [2e-12, 0.034]	50.0% [0.0, 90.8]	0.025 [0.000, 0.104]	[-0.288, 0.148]
SEEK Visual	FSIQ	835	-0.044 [-0.138, 0.050]	0.878	-3.631	ZERO++	0.004 [3e-14, 0.032]	64.1% [0.0, 93.2]	0.042 [0.001, 0.109]	[-0.258, 0.169]
SEEK Tactile	FSIQ	620	-0.079 [-0.186, 0.041]	0.649	-2.275	ZERO+	0.003 [1e-13, 0.032]	46.3% [0.0, 90.4]	0.069 [0.009, 0.186]	[-0.283, 0.137]
SEEK Oral Tactile	FSIQ	846	0.026 [-0.068, 0.117]	0.941	-4.423	ZERO++	0.003 [2e-12, 0.031]	60.3% [0.0, 93.2]	0.058 [0.010, 0.142]	[-0.184, 0.228]
SEEK Movement	FSIQ	852	0.011 [-0.074, 0.096]	0.975	-5.311	ZERO++	0.002 [5e-12, 0.018]	47.3% [0.0, 90.5]	0.032 [0.000, 0.094]	[-0.148, 0.178]
**Correlations with VABS Communication**										
**Sensory Construct**	**Correlate**	***n***	***r* [95% CrI]**	***P*ROPE**	**log(*BF*ROPE)**	**Sig***	**τ² [95% CrI]**	***I*² [95% CrI]**	**ICC [95% CrI]**	**95% PI for *r***
HYPER General	VABS COM	1590	-0.091 [-0.228, 0.035]	0.554	-1.877	ZERO+	0.036 [2e-10, 0.115]	88.5% [51.0, 98.7]	0.105 [0.033, 0.207]	[-0.506, 0.314]
HYPER Auditory	VABS COM	1590	-0.053 [-0.186, 0.072]	0.769	-2.867	ZERO++	0.031 [3e-11, 0.120]	88.0% [23.5, 98.9]	0.077 [0.015, 0.170]	[-0.459, 0.360]
HYPER Visual	VABS COM	1455	-0.016 [-0.145, 0.092]	0.887	-3.727	ZERO++	0.014 [2e-12, 0.079]	79.2% [5.2, 98.2]	0.093 [0.027, 0.204]	[-0.359, 0.298]
HYPER Tactile	VABS COM	1581	-0.065 [-0.177, 0.047]	0.750	-2.755	ZERO++	0.021 [6e-11, 0.080]	86.8% [13.4, 98.7]	0.084 [0.023, 0.173]	[-0.411, 0.281]
HYPER Gustatory	VABS COM	1444	-0.001 [-0.115, 0.099]	0.936	-4.343	ZERO++	0.009 [1e-11, 0.064]	76.5% [2.4, 98.0]	0.071 [0.018, 0.160]	[-0.303, 0.283]
HYPER Olfactory	VABS COM	640	0.092 [-0.331, 0.509]	0.385	-1.196	ZERO+	0.075 [2e-10, 1.620]	61.1% [0.0, 97.1]	0.503 [0.078, 0.939]	[-0.726, 0.850]
HYPER Movement	VABS COM	1392	-0.057 [-0.195, 0.051]	0.755	-2.778	ZERO++	0.017 [4e-11, 0.092]	81.1% [10.2, 98.6]	0.068 [0.012, 0.172]	[-0.417, 0.281]
HYPO Speech	VABS COM	1589	-0.081 [-0.204, 0.013]	0.630	-2.206	ZERO+	0.013 [1e-10, 0.065]	80.1% [3.9, 97.6]	0.084 [0.027, 0.167]	[-0.401, 0.204]
HYPO Pain/Temp	VABS COM	1280	-0.195 [-0.326, -0.063]	0.070	0.933	INC	0.018 [2e-09, 0.074]	78.6% [41.0, 97.6]	0.161 [0.051, 0.331]	[-0.508, 0.137]
SEEK Visual	VABS COM	1286	-0.119 [-0.252, 0.040]	0.386	-1.201	ZERO+	0.019 [3e-09, 0.102]	76.8% [27.6, 98.4]	0.061 [0.009, 0.173]	[-0.457, 0.273]
SEEK Tactile	VABS COM	1571	-0.141 [-0.272, -0.029]	0.210	-0.325	INC	0.014 [5e-13, 0.064]	77.7% [7.3, 97.4]	0.056 [0.012, 0.127]	[-0.455, 0.153]
SEEK Oral Tactile	VABS COM	1236	-0.167 [-0.296, -0.043]	0.122	0.314	INC	0.013 [3e-10, 0.075]	74.4% [8.4, 98.3]	0.066 [0.009, 0.182]	[-0.477, 0.138]
SEEK Movement	VABS COM	1574	-0.086 [-0.160, -0.008]	0.651	-2.276	ZERO+	0.004 [5e-12, 0.023]	71.8% [4.2, 97.4]	0.084 [0.031, 0.167]	[-0.264, 0.104]

Table continues on following page.

Supplemental Table S6 (Continued: Page 9 of 10)

Correlations with VABS Daily Living Skills

Sensory Construct	Correlate	*n*	*r* [95% CrI]	P_{ROPE}	log(BF_{ROPE})	Sig*	τ^2 [95% CrI]	I^2 [95% CrI]	ICC [95% CrI]	95% PI for *r*
HYPER General	VABS DLS	1449	-0.092 [-0.195, -0.001]	0.571	-1.946	ZERO+	0.009 [2e-12, 0.048]	77.7% [5.8, 98.5]	0.093 [0.027, 0.204]	[-0.347, 0.172]
HYPER Auditory	VABS DLS	1449	-0.069 [-0.166, 0.025]	0.764	-2.837	ZERO++	0.006 [3e-12, 0.054]	71.4% [0.0, 95.9]	0.072 [0.011, 0.192]	[-0.330, 0.190]
HYPER Visual	VABS DLS	1445	-0.035 [-0.136, 0.057]	0.901	-3.875	ZERO++	0.009 [8e-12, 0.044]	78.0% [9.0, 98.2]	0.086 [0.028, 0.182]	[-0.300, 0.210]
HYPER Tactile	VABS DLS	1440	-0.095 [-0.174, -0.015]	0.554	-1.890	ZERO+	0.003 [4e-13, 0.026]	62.0% [0.0, 93.9]	0.082 [0.020, 0.196]	[-0.283, 0.084]
HYPER Gustatory	VABS DLS	1433	-0.031 [-0.119, 0.056]	0.942	-4.464	ZERO++	0.004 [6e-13, 0.035]	65.3% [0.0, 94.7]	0.067 [0.016, 0.160]	[-0.247, 0.185]
HYPER Olfactory	VABS DLS	639	0.090 [-0.376, 0.531]	0.363	-1.091	INC	0.103 [4e-11, 1.877]	64.2% [0.0, 97.0]	0.551 [0.103, 0.960]	[-0.724, 0.908]
HYPER Movement	VABS DLS	1382	-0.123 [-0.228, -0.031]	0.312	-0.871	INC	0.009 [4e-14, 0.045]	77.9% [10.8, 98.0]	0.062 [0.011, 0.154]	[-0.385, 0.127]
HYPO Speech	VABS DLS	1448	-0.075 [-0.174, 0.016]	0.715	-2.586	ZERO++	0.006 [6e-12, 0.045]	71.4% [0.0, 95.1]	0.090 [0.026, 0.199]	[-0.325, 0.162]
HYPO Pain/Temp	VABS DLS	1277	-0.164 [-0.284, -0.047]	0.127	0.252	INC	0.014 [3e-10, 0.062]	78.5% [31.4, 98.1]	0.165 [0.055, 0.342]	[-0.455, 0.137]
SEEK Visual	VABS DLS	1283	-0.117 [-0.230, 0.019]	0.382	-1.190	ZERO+	0.012 [2e-13, 0.070]	74.6% [4.8, 97.8]	0.063 [0.010, 0.164]	[-0.410, 0.206]
SEEK Tactile	VABS DLS	1437	-0.106 [-0.186, -0.013]	0.443	-1.440	ZERO+	0.002 [1e-14, 0.022]	51.6% [0.0, 91.8]	0.055 [0.011, 0.130]	[-0.267, 0.086]
**SEEK Oral Tactile**	**VABS DLS**	**1234**	**-0.187 [-0.291, -0.070]**	**0.059**	**1.123**	**SIG+**	**0.006 [2e-12, 0.055]**	**65.6% [0.0, 94.3]**	**0.067 [0.007, 0.193]**	**[-0.440, 0.072]**
SEEK Movement	VABS DLS	1434	-0.063 [-0.136, 0.014]	0.854	-3.419	ZERO++	0.002 [1e-13, 0.016]	53.8% [0.0, 91.6]	0.099 [0.034, 0.208]	[-0.206, 0.099]

Correlations with VABS Socialization

Sensory Construct	Correlate	*n*	*r* [95% CrI]	P_{ROPE}	log(BF_{ROPE})	Sig*	τ^2 [95% CrI]	I^2 [95% CrI]	ICC [95% CrI]	95% PI for *r*
HYPER General	VABS SOC	1589	-0.120 [-0.229, -0.018]	0.342	-1.011	INC	0.017 [5e-12, 0.069]	84.9% [34.0, 98.7]	0.087 [0.029, 0.180]	[-0.430, 0.197]
HYPER Auditory	VABS SOC	1589	-0.078 [-0.195, 0.033]	0.665	-2.333	ZERO++	0.018 [1e-11, 0.093]	84.5% [7.4, 98.9]	0.071 [0.014, 0.165]	[-0.427, 0.276]
HYPER Visual	VABS SOC	1455	-0.042 [-0.149, 0.055]	0.853	-3.406	ZERO++	0.007 [1e-12, 0.043]	70.9% [0.0, 93.9]	0.085 [0.027, 0.182]	[-0.299, 0.193]
HYPER Tactile	VABS SOC	1580	-0.122 [-0.236, -0.026]	0.306	-0.847	INC	0.009 [4e-13, 0.060]	76.5% [3.4, 98.0]	0.080 [0.022, 0.177]	[-0.406, 0.157]
HYPER Gustatory	VABS SOC	1443	-0.045 [-0.171, 0.059]	0.834	-3.281	ZERO++	0.012 [2e-12, 0.083]	78.5% [2.9, 98.4]	0.075 [0.019, 0.170]	[-0.375, 0.274]
HYPER Olfactory	VABS SOC	640	0.087 [-0.392, 0.553]	0.369	-1.131	ZERO+	0.119 [2e-11, 2.213]	65.4% [0.0, 97.2]	0.520 [0.092, 0.936]	[-0.757, 0.914]
HYPER Movement	VABS SOC	1391	-0.088 [-0.211, 0.017]	0.580	-1.979	ZERO+	0.013 [4e-11, 0.061]	78.1% [7.5, 98.0]	0.062 [0.013, 0.151]	[-0.397, 0.196]
HYPO Speech	VABS SOC	1588	-0.163 [-0.289, -0.050]	0.135	0.193	INC	0.027 [9e-12, 0.092]	86.7% [46.1, 98.6]	0.105 [0.039, 0.202]	[-0.516, 0.206]
**HYPO Pain/Temp**	**VABS SOC**	**1279**	**-0.182 [-0.267, -0.091]**	**0.038**	**1.555**	**SIG+**	**0.002 [1e-13, 0.028]**	**50.8% [0.0, 92.8]**	**0.157 [0.045, 0.332]**	**[-0.361, 0.006]**
SEEK Visual	VABS SOC	1285	-0.144 [-0.275, -0.007]	0.237	-0.491	INC	0.009 [5e-11, 0.065]	65.1% [0.0, 93.3]	0.052 [0.008, 0.142]	[-0.419, 0.174]
SEEK Tactile	VABS SOC	1570	-0.136 [-0.251, -0.037]	0.225	-0.416	INC	0.014 [2e-11, 0.065]	81.5% [5.2, 98.1]	0.057 [0.011, 0.132]	[-0.444, 0.159]
SEEK Oral Tactile	VABS SOC	1235	-2e-01 [-3e-01, -9e-04]	0.188	-0.209	INC	0.026 [1e-11, 0.158]	79.5% [12.0, 98.9]	0.093 [0.012, 0.240]	[-0.575, 0.273]
SEEK Movement	VABS SOC	1573	-0.119 [-0.194, -0.043]	0.307	-0.839	INC	0.002 [1e-12, 0.023]	52.8% [0.0, 93.7]	0.087 [0.032, 0.174]	[-0.282, 0.055]

Table continues on following page.

Supplemental Table S6 (Continued: Page 10 of 10)

**Correlations with VABS Adaptive Behavior Composite**										
**Sensory Construct**	**Correlate**	***n***	***r* [95% CrI]**	***P*ROPE**	**log(*BF*ROPE)**	**Sig***	**τ^2 [95% CrI]**	**I^2 [95% CrI]**	**ICC [95% CrI]**	**95% PI for *r***
HYPER General	VABS ABC	1472	-0.111 [-0.245, 0.003]	0.422	-1.357	ZERO+	0.022 [4e-10, 0.097]	83.6% [24.8, 98.9]	0.096 [0.029, 0.202]	[-0.481, 0.239]
HYPER Auditory	VABS ABC	1472	-0.093 [-0.248, 0.049]	0.535	-1.788	ZERO+	0.046 [9e-11, 0.169]	88.8% [33.9, 99.1]	0.098 [0.022, 0.215]	[-0.548, 0.388]
HYPER Visual	VABS ABC	1339	-0.009 [-0.116, 0.085]	0.940	-4.408	ZERO++	0.004 [1e-11, 0.041]	61.7% [0.0, 94.0]	0.085 [0.026, 0.189]	[-0.260, 0.205]
HYPER Tactile	VABS ABC	1463	-0.111 [-0.228, -0.005]	0.404	-1.270	ZERO+	0.013 [8e-12, 0.073]	79.7% [4.9, 98.3]	0.089 [0.023, 0.197]	[-0.422, 0.202]
HYPER Gustatory	VABS ABC	1327	-0.027 [-0.162, 0.091]	0.866	-3.539	ZERO++	0.015 [3e-12, 0.098]	78.8% [4.3, 98.7]	0.082 [0.020, 0.189]	[-0.384, 0.317]
HYPER Olfactory	VABS ABC	640	0.093 [-0.362, 0.519]	0.376	-1.178	ZERO+	0.078 [2e-10, 1.606]	59.1% [0.0, 96.7]	0.519 [0.076, 0.941]	[-0.701, 0.885]
HYPER Movement	VABS ABC	1277	-0.097 [-0.232, 0.025]	0.521	-1.735	ZERO+	0.019 [6e-10, 0.091]	81.1% [17.0, 98.5]	0.082 [0.016, 0.200]	[-0.454, 0.250]
HYPO Speech	VABS ABC	1472	-0.130 [-0.273, -0.010]	0.319	-0.894	INC	0.028 [2e-11, 0.109]	84.9% [17.2, 98.6]	0.114 [0.037, 0.227]	[-0.530, 0.238]
**HYPO Pain/Temp**	**VABS ABC**	**1164**	**-0.199 [-0.310, -0.082]**	**0.043**	**1.427**	**SIG+**	**0.009 [1e-11, 0.051]**	**70.4% [6.5, 97.7]**	**0.173 [0.052, 0.362]**	**[-0.449, 0.070]**
SEEK Visual	VABS ABC	1170	-0.150 [-0.281, 0.013]	0.241	-0.518	INC	0.016 [2e-11, 0.100]	73.2% [6.9, 98.2]	0.076 [0.011, 0.208]	[-0.473, 0.235]
SEEK Tactile	VABS ABC	1453	-0.147 [-0.259, -0.046]	0.155	0.039	INC	0.009 [4e-13, 0.063]	75.4% [0.0, 95.4]	0.060 [0.013, 0.139]	[-0.428, 0.146]
SEEK Oral Tactile	VABS ABC	1121	-0.180 [-0.335, -0.018]	0.132	0.208	INC	0.022 [1e-13, 0.143]	76.2% [8.3, 98.9]	0.097 [0.012, 0.256]	[-0.565, 0.234]
SEEK Movement	VABS ABC	1456	-0.109 [-0.183, -0.035]	0.399	-1.268	ZERO+	0.002 [1e-13, 0.018]	51.3% [0.0, 92.6]	0.097 [0.035, 0.194]	[-0.260, 0.047]

Correlation values are derived from random-effects integrative data analysis (IDA) models (see Supplemental Table S3 for full model specification and calculation of the effect size parameter). Practically significant effects (log(BF_{ROPE}) > 1.1) are colored blue (positive effects) or orange (negative effects) and bolded, and trivially small effects (i.e., log(BF_{ROPE}) < -1.1) are highlighted in gray. Inconclusive effects (-1.1 < BF_{ROPE} < 1.1) are colored yellow. **CrI** = Bayesian highest-density credible interval; $\boldsymbol{P_{ROPE}}$ = posterior probability that the effect size (*r*) falls within [-.1, .1]; **log(BF_{ROPE})** = natural logarithm of the ROPE Bayes factor; $\boldsymbol{\tau^2}$ = raw heterogeneity (on scale of beta coefficient); $\boldsymbol{I^2}$ = standardized heterogeneity coefficient (presented as percentage); **ICC** = intraclass correlation (proportion of outcome variance explained by random slope/intercept terms); **PI** = prediction interval; **HYPER** = hyperreactivity; **HYPO** = hyporeactivity; **SEEK** = sensory seeking; **VIQ** = verbal IQ; **NVIQ** = nonverbal IQ; **FSIQ** = full-scale IQ; **DQ** = developmental quotient; **VABS** = Vineland Adaptive Behavior Scales (Sparrow, 2011); **COM** = VABS Communication; **DLS** = VABS Daily Living Skills; **SOC** = VABS Socialization; **ABC** = VABS Adaptive Behavior Composite; **PSYC** = psychiatric symptoms as measured by the Achenbach System of Empirically Based Assessment (Achenbach, 2009), a version of the Behavior Assessment System for Children (Reynolds & Kamphaus, 2015), or the Conners-3 (Conners, 2008); **INT** = internalizing symptoms; **EXT** = externalizing symptoms; **ADHD** = attention deficit hyperactivity disorder symptoms; **SRS** = Social Responsiveness Scale (Constantino et al., 2003; Constantino & Gruber, 2012); **RBS-R** = Repetitive Behavior Scale–Revised (Bodfish et al., 2000); **RSM** = repetitive sensory motor (stereotypy); **SIB** = self-injurious behavior; **RSC** = ritualistic/sameness and compulsive behavior.

* Significance levels are derived from values of BF_{ROPE} and an interval null hypothesis (ROPE) of *r* = [-.l, .1]: **ZERO++** = strong evidence for null hypothesis (log(BF_{ROPE}) < -2.3); **ZERO+** = moderate evidence for null hypothesis (log(BF_{ROPE}) < -1.1); **SIG++** = strong evidence for alternative hypothesis (log(BF_{ROPE}) > 2.3); **SIG+** = moderate evidence for alternative hypothesis (log(BF_{ROPE}) > 1.1); **INC** = inconclusive evidence for or against null hypothesis (-1.1 < BF_{ROPE} < 1.1).

Supplemental Table S7

Meta-analytic summary effects and heterogeneity parameters for binary correlates

Sensory Construct	Correlate	*n*	*d* [95% CrI]	P_{ROPE}	log(BF_{ROPE})	Sig*	τ^2 [95% CrI]	I^2 [95% CrI]	ICC [95% CrI]	95% PI for *d*
HYPER General	Sex (M > F)	3865	0.008 [-0.122, 0.132]	0.996	-6.526	ZERO++	0.004 [8e-06, 0.049]	50.5% [0.0, 90.5]	0.078 [0.029, 0.154]	[-0.253, 0.245]
HYPER Auditory	Sex (M > F)	3859	0.009 [-0.115, 0.128]	0.997	-6.742	ZERO++	0.006 [1e-05, 0.068]	61.3% [0.0, 93.3]	0.044 [0.010, 0.111]	[-0.281, 0.277]
HYPER Visual	Sex (M > F)	3699	-0.010 [-0.128, 0.105]	0.997	-6.664	ZERO++	0.003 [6e-06, 0.048]	48.6% [0.0, 91.2]	0.095 [0.037, 0.185]	[-0.250, 0.210]
HYPER Tactile	Sex (M > F)	3836	-0.037 [-0.177, 0.093]	0.984	-5.096	ZERO++	0.006 [1e-05, 0.072]	58.5% [0.0, 92.6]	0.087 [0.029, 0.176]	[-0.352, 0.240]
HYPER Gustatory	Sex (M > F)	3681	0.162 [0.036, 0.294]	0.724	-1.955	ZERO+	0.003 [7e-06, 0.045]	44.9% [0.0, 89.0]	0.050 [0.015, 0.120]	[-0.070, 0.400]
HYPER Olfactory	Sex (M > F)	2467	-0.063 [-0.466, 0.299]	0.799	-2.372	ZERO++	0.023 [5e-05, 0.969]	41.4% [0.0, 94.3]	0.188 [0.002, 0.679]	[-0.902, 0.740]
HYPER Movement	Sex (M > F)	3523	0.071 [-0.065, 0.214]	0.961	-4.193	ZERO++	0.004 [9e-06, 0.061]	47.5% [0.0, 90.3]	0.036 [0.006, 0.103]	[-0.180, 0.354]
HYPO Speech	Sex (M > F)	3854	0.066 [-0.058, 0.186]	0.987	-5.297	ZERO++	0.004 [7e-06, 0.049]	49.8% [0.0, 90.8]	0.064 [0.021, 0.138]	[-0.179, 0.302]
HYPO Pain/Temp	Sex (M > F)	3483	-0.054 [-0.182, 0.077]	0.982	-5.008	ZERO++	0.003 [7e-06, 0.065]	47.7% [0.0, 92.0]	0.156 [0.060, 0.299]	[-0.317, 0.213]
SEEK Visual	Sex (M > F)	3501	0.084 [-0.132, 0.250]	0.921	-3.434	ZERO++	0.014 [5e-05, 0.119]	61.9% [0.0, 91.2]	0.067 [0.015, 0.170]	[-0.362, 0.441]
SEEK Tactile	Sex (M > F)	3815	-0.043 [-0.166, 0.090]	0.992	-5.808	ZERO++	0.004 [8e-06, 0.064]	51.9% [0.0, 92.2]	0.044 [0.009, 0.110]	[-0.313, 0.224]
SEEK Oral Tactile	Sex (M > F)	3366	-0.068 [-0.254, 0.082]	0.916	-3.366	ZERO++	0.008 [2e-05, 0.097]	54.4% [0.0, 91.4]	0.060 [0.011, 0.165]	[-0.448, 0.241]
SEEK Movement	Sex (M > F)	3823	0.031 [-0.137, 0.183]	0.978	-4.786	ZERO++	0.016 [3e-05, 0.167]	73.9% [0.0, 95.7]	0.101 [0.025, 0.270]	[-0.432, 0.473]
HYPER General	ID Status	1030	-0.059 [-0.360, 0.242]	0.784	-2.268	ZERO+	0.018 [4e-05, 0.266]	47.4% [0.0, 90.3]	0.067 [0.003, 0.220]	[-0.640, 0.515]
HYPER Auditory	ID Status	1029	-0.131 [-0.421, 0.153]	0.678	-1.721	ZERO+	0.022 [5e-05, 0.340]	54.6% [0.0, 92.6]	0.058 [0.000, 0.235]	[-0.765, 0.466]
HYPER Visual	ID Status	959	-0.134 [-0.397, 0.140]	0.683	-1.754	ZERO+	0.012 [3e-05, 0.224]	43.5% [0.0, 90.4]	0.083 [0.013, 0.221]	[-0.630, 0.387]
HYPER Tactile	ID Status	1026	-0.024 [-0.334, 0.284]	0.814	-2.445	ZERO++	0.025 [5e-05, 0.523]	54.0% [0.0, 94.3]	0.068 [0.000, 0.313]	[-0.746, 0.684]
HYPER Gustatory	ID Status	956	-0.071 [-0.402, 0.228]	0.771	-2.199	ZERO+	0.038 [8e-05, 0.452]	64.2% [0.0, 94.0]	0.067 [0.000, 0.299]	[-0.846, 0.648]
HYPER Olfactory	ID Status	109	-0.258 [-1.443, 0.849]	0.297	-0.114	INC	0.345 [6e-04, 9.365]	58.7% [0.0, 96.1]	0.515 [0.051, 0.939]	[-3.319, 2.616]
HYPER Movement	ID Status	887	0.134 [-0.248, 0.481]	0.615	-1.455	ZERO+	0.065 [2e-04, 0.740]	69.4% [0.0, 95.0]	0.102 [0.000, 0.410]	[-0.838, 1.054]
HYPO Speech	ID Status	1027	-0.148 [-0.435, 0.131]	0.640	-1.546	ZERO+	0.023 [6e-05, 0.337]	58.1% [0.0, 93.4]	0.115 [0.030, 0.300]	[-0.811, 0.471]
HYPO Pain/Temp	ID Status	854	0.415 [0.035, 0.784]	0.117	1.044	INC	0.078 [9e-04, 0.519]	73.3% [9.8, 97.7]	0.211 [0.066, 0.431]	[-0.487, 1.309]
SEEK Visual	ID Status	855	0.359 [-0.135, 0.762]	0.216	0.308	INC	0.063 [2e-04, 1.095]	59.6% [0.0, 94.5]	0.100 [0.000, 0.484]	[-0.754, 1.384]
SEEK Tactile	ID Status	1013	0.127 [-0.178, 0.429]	0.675	-1.728	ZERO+	0.038 [9e-05, 0.402]	64.8% [0.0, 93.6]	0.090 [0.009, 0.287]	[-0.582, 0.849]
**SEEK Oral Tactile**	**ID Status**	**797**	**0.422 [0.073, 0.768]**	**0.092**	**1.305**	**SIG+**	**0.026 [6e-05, 0.467]**	**51.7% [0.0, 92.8]**	**0.093 [0.010, 0.317]**	**[-0.314, 1.149]**
SEEK Movement	ID Status	1024	0.087 [-0.222, 0.363]	0.763	-2.162	ZERO+	0.035 [9e-05, 0.355]	65.0% [0.0, 93.4]	0.091 [0.011, 0.289]	[-0.621, 0.749]

Correlation values are derived from random-effects integrative data analysis (IDA) models (see Supplemental Table S3 for full model specification and calculation of the effect size parameter). Practically significant effects (log(BF_{ROPE}) > 1.1) are colored blue (positive effects) or orange (negative effects) and bolded, and trivially small effects (log(BF_{ROPE}) < -1.1) are highlighted in gray. Inconclusive effects (-1.1 < log(BF_{ROPE}) < 1.1) are colored yellow. **CrI** = Bayesian highest-density credible interval; $\mathbf{P_{ROPE}}$ = posterior probability that the effect size (*d*) falls within [-0.2, 0.2]; **log(BF_{ROPE})** = natural logarithm of the ROPE Bayes factor; $\boldsymbol{\tau^2}$ = raw heterogeneity (on scale of beta coefficient); $\boldsymbol{I^2}$ = standardized heterogeneity coefficient; **ICC** = intraclass correlation (proportion of outcome variance explained by random slope/intercept terms); **PI** = prediction interval; **HYPER** = hyperreactivity; **HYPO** = hyporeactivity; **SEEK** = sensory seeking; **ID** = intellectual disability.
* Significance levels are derived from values of BF_{ROPE} and an interval null hypothesis (ROPE) of *d* = [-0.2, 0.2]: **ZERO++** = strong evidence for null hypothesis (log(BF_{ROPE}) < -2.3); **ZERO+** = moderate evidence for null hypothesis (log(BF_{ROPE}) < -1.1); **SIG++** = strong evidence for alternative hypothesis (log(BF_{ROPE}) > 2.3); **SIG+** = moderate evidence for alternative hypothesis (log(BF_{ROPE}) > 1.1); **INC** = inconclusive evidence for or against null hypothesis (-1.1 < BF_{ROPE} < 1.1).

Supplemental References

Achenbach, T. M. (2009). *Achenbach System of Empirically Based Assessment (ASEBA): Development, findings, theory, and applications*. University of Vermont, Research Center of Children, Youth & Families.

Ausderau, K. K., Sideris, J., Furlong, M., Little, L. M., Bulluck, J., & Baranek, G. T. (2014). National survey of sensory features in children with ASD: Factor structure of the Sensory Experience Questionnaire (3.0). *Journal of Autism and Developmental Disorders*, *44*(4), 915–925. https://doi.org/10.1007/s10803-013-1945-1

Baranek, G. T. (1999). *Sensory Experiences Questionnaire (SEQ), version 2.1* [Questionnaire]. University of North Carolina at Chapel Hill.

Baranek, G. T. (2009). *Sensory Experiences Questionnaire (SEQ), version 3.0* [Questionnaire]. University of North Carolina at Chapel Hill.

Baranek, G. T., David, F. J., Poe, M. D., Stone, W. L., & Watson, L. R. (2006). Sensory Experiences Questionnaire: Discriminating sensory features in young children with autism, developmental delays, and typical development. *Journal of Child Psychology and Psychiatry*, *47*(6), 591–601. https://doi.org/10.1111/j.1469-7610.2005.01546.x

Bishop, S. L., Hus, V., Duncan, A., Huerta, M., Gotham, K., Pickles, A., Kreiger, A., Buja, A., Lund, S., & Lord, C. (2013). Subcategories of restricted and repetitive behaviors in children with autism spectrum disorders. *Journal of Autism and Developmental Disorders*, *43*(6), 1287–1297. https://doi.org/10.1007/s10803-012-1671-0

Bock, R. D., & Aitkin, M. (1981). Marginal maximum likelihood estimation of item parameters: Application of an EM algorithm. *Psychometrika*, *46*(4), 443–459. https://doi.org/10.1007/bf02293801

Bodfish, J. W., Symons, F. J., Parker, D. E., & Lewis, M. H. (2000). Varieties of repetitive behavior in autism: Comparisons to mental retardation. *Journal of Autism and Developmental Disorders, 30*(3), 237–243. https://doi.org/10.1023/A:1005596502855

Boyd, B. A., Baranek, G. T., Sideris, J., Poe, M. D., Watson, L. R., Patten, E., & Miller, H. (2010). Sensory features and repetitive behaviors in children with autism and developmental delays. *Autism Research*, *3*(2), 78–87. https://doi.org/10.1002/aur.124

Burns, C. O., Dixon, D. R., Novack, M., & Granpeesheh, D. (2017). A systematic review of assessments for sensory processing abnormalities in autism spectrum disorder. *Review Journal of Autism and Developmental Disorders*, *4*(3), 209–224. https://doi.org/10.1007/s40489-017-0109-1

Cai, L., Chung, S. W., & Lee, T. (2023). Incremental model fit assessment in the case of categorical data: Tucker–Lewis index for item response theory modeling. *Prevention Science*, *24*(3), 455–466. https://doi.org/10.1007/s11121-021-01253-4

Cai, L., & Monroe, S. (2014). *A new statistic for evaluating item response theory models for ordinal data* (CRESST Report 839; pp. 1–28). University of California, National Center for Research on Evaluation, Standards, and Student Testing (CRESST). https://eric.ed.gov/?id=ED555726

Cascio, C. J., Gu, C., Schauder, K. B., Key, A. P., & Yoder, P. (2015). Somatosensory event-related potentials and association with tactile behavioral responsiveness patterns in children with ASD. *Brain Topography*, *28*(6), 895–903. https://doi.org/10.1007/s10548-015-0439-1

Cascio, C. J., Lorenzi, J., & Baranek, G. T. (2016). Self-reported pleasantness ratings and examiner-coded defensiveness in response to touch in children with ASD: Effects of

stimulus material and bodily location. *Journal of Autism and Developmental Disorders*, *46*(5), 1528–1537. https://doi.org/10.1007/s10803-013-1961-1

Chalmers, R. P. (2012). mirt: A multidimensional item response theory package for the R environment. *Journal of Statistical Software*, *48*(6), 1–29. https://doi.org/10.18637/jss.v048.i06

Christensen, A. P., Garrido, L. E., Guerra-Peña, K., & Golino, H. (2023). Comparing community detection algorithms in psychometric networks: A Monte Carlo simulation. *Behavior Research Methods*, Advance online publication. https://doi.org/10.3758/s13428-023-02106-4

Conners, C. K. (2008). *Conners 3* (3rd ed.). Multi-Health Systems.

Constantino, J. N., Davis, S. A., Todd, R. D., Schindler, M. K., Gross, M. M., Brophy, S. L., Metzger, L. M., Shoushtari, C. S., Splinter, R., & Reich, W. (2003). Validation of a brief quantitative measure of autistic traits: Comparison of the Social Responsiveness Scale with the Autism Diagnostic Interview-Revised. *Journal of Autism and Developmental Disorders*, *33*(4), 427–433. https://doi.org/10.1023/A:1025014929212

Constantino, J. N., & Gruber, C. P. (2012). *Social Responsiveness Scale–Second Edition (SRS-2): Manual* (2nd ed.). Western Psychological Services.

Cooksey, R. W., & Soutar, G. N. (2006). Coefficient beta and hierarchical item clustering: An analytical procedure for establishing and displaying the dimensionality and homogeneity of summated scales. *Organizational Research Methods*, *9*(1), 78–98. https://doi.org/10.1177/1094428105283939

Donkers, F. C. L., Schipul, S. E., Baranek, G. T., Cleary, K. M., Willoughby, M. T., Evans, A. M., Bulluck, J. C., Lovmo, J. E., & Belger, A. (2015). Attenuated auditory event-related potentials and associations with atypical sensory response patterns in children with

autism. *Journal of Autism and Developmental Disorders*, *45*(2), 506–523. https://doi.org/10.1007/s10803-013-1948-y

Dunn, W. (1999). *Sensory Profile: User's manual*. Psychological Corporation.

Dunn, W. (2014). *Sensory Profile 2: User's manual*. Pearson.

Dwyer, P., Ferrer, E., Saron, C. D., & Rivera, S. M. (2022). Exploring sensory subgroups in typical development and autism spectrum development using factor mixture modelling. *Journal of Autism and Developmental Disorders*, *52*(9), 3840–3860. https://doi.org/10.1007/s10803-021-05256-6

Elliott, C. D. (2007). *Differential Ability Scales* (2nd ed.). Pearson/Psychological Corp.

Feldman, J. I., Cassidy, M., Liu, Y., Kirby, A. V., Wallace, M. T., & Woynaroski, T. G. (2020). Relations between sensory responsiveness and features of autism in children. *Brain Sciences*, *10*(11), Article 11. https://doi.org/10.3390/brainsci10110775

Feldman, J. I., Raj, S., Bowman, S. M., Santapuram, P., Golden, A. J., Daly, C., Dunham, K., Suzman, E., Augustine, A. E., Garla, V., Muhumuza, A., Cascio, C. J., Williams, K. L., Kirby, A. V., Keceli, -Kaysili Bahar, & Woynaroski, T. G. (2021). Sensory responsiveness is linked with communication in infant siblings of children with and without autism. *Journal of Speech, Language, and Hearing Research*, *64*(6), 1964–1976. https://doi.org/10.1044/2021_JSLHR-20-00196

Foss-Feig, J. H., Heacock, J. L., & Cascio, C. J. (2012). Tactile responsiveness patterns and their association with core features in autism spectrum disorders. *Research in Autism Spectrum Disorders*, *6*(1), 337–344. https://doi.org/10.1016/j.rasd.2011.06.007

Gibbons, R. D., Bock, R. D., Hedeker, D., Weiss, D. J., Segawa, E., Bhaumik, D. K., Kupfer, D. J., Frank, E., Grochocinski, V. J., & Stover, A. (2007). Full-information item bifactor

analysis of graded response data. *Applied Psychological Measurement*, *31*(1), 4–19. https://doi.org/10.1177/0146621606289485

Golino, H. F., & Christensen, A. (2020). *EGAnet: Exploratory Graph Analysis—A Framework for Estimating the Number of Dimensions in Multivariate Data Using Network Psychometrics*. https://CRAN.R-project.org/package=EGAnet

Golino, H. F., Shi, D., Christensen, A. P., Garrido, L. E., Nieto, M. D., Sadana, R., Thiyagarajan, J. A., & Martinez-Molina, A. (2020). Investigating the performance of exploratory graph analysis and traditional techniques to identify the number of latent factors: A simulation and tutorial. *Psychological Methods*, *25*(3), 292–320. https://doi.org/10.1037/met0000255

Green, S. B., & Yang, Y. (2009). Reliability of summed item scores using structural equation modeling: An alternative to coefficient alpha. *Psychometrika*, *74*(1), 155–167. https://doi.org/10.1007/s11336-008-9099-3

Lane, A. E., Simpson, K., Masi, A., Grove, R., Moni, M. A., Montgomery, A., Roberts, J., Silove, N., Whalen, O., Whitehouse, A. J. O., & Eapen, V. (2022). Patterns of sensory modulation by age and sex in young people on the autism spectrum. *Autism Research*, *15*(10), 1840–1854. https://doi.org/10.1002/aur.2762

Lee, H., Chen, Y.-J., Sideris, J., Watson, L. R., Crais, E. R., & Baranek, G. T. (2022). Sensory features of young children from a large community sample: Latent factor structures of the Sensory Experiences Questionnaire (Version 2.1, Short Form). *The American Journal of Occupational Therapy*, *76*(3), Article 7603205140. https://doi.org/10.5014/ajot.2022.046995

Little, L. M., Freuler, A. C., Houser, M. B., Guckian, L., Carbine, K., David, F. J., & Baranek, G. T. (2011). Psychometric validation of the Sensory Experiences Questionnaire. *The*

American Journal of Occupational Therapy, *65*(2), 207–210. https://doi.org/10.5014/ajot.2011.000844

Maydeu-Olivares, A. (2013). Goodness-of-fit assessment of item response theory models. *Measurement: Interdisciplinary Research and Perspectives*, *11*(3), 71–101. https://doi.org/10.1080/15366367.2013.831680

Maydeu-Olivares, A., & Joe, H. (2014). Assessing approximate fit in categorical data analysis. *Multivariate Behavioral Research*, *49*(4), 305–328. https://doi.org/10.1080/00273171.2014.911075

McIntosh, D. N., Miller, L. J., & Shyu, V. (1999). Development and validation of the Short Sensory Profile. In W. Dunn (Ed.), *Sensory Profile manual* (pp. 59–73). Psychological Corporation.

Monroe, S., & Cai, L. (2015). Evaluating structural equation models for categorical outcomes: A new test statistic and a practical challenge of interpretation. *Multivariate Behavioral Research*, *50*(6), 569–583. https://doi.org/10.1080/00273171.2015.1032398

Mullen, E. M. (1995). *Mullen Scales of Early Learning*. American Guidance Service.

Nowell, K. P., Bernardin, C. J., Brown, C., & Kanne, S. (2021). Characterization of special interests in autism spectrum disorder: A brief review and pilot study using the Special Interests Survey. *Journal of Autism and Developmental Disorders*, *51*(8), 2711–2724. https://doi.org/10.1007/s10803-020-04743-6

Parks, K., Schulz, S., McDonnell, C. G., Anagnostou, E., Nicolson, R., Kelley, E., Georgiades, S., Crosbie, J., Schachar, R., Liu, X., & Stevenson, R. (2020). *Sensory processing in ASD and ADHD: A confirmatory factor analysis*. PsyArXiv. https://doi.org/10.31234/osf.io/myjbq

Revelle, W. (1978). ICLUST: A cluster analytic approach to exploratory and confirmatory scale construction. *Behavior Research Methods & Instrumentation*, *10*(5), 739–742. https://doi.org/10.3758/BF03205389

Revelle, W. (2022). *psych: Procedures for Psychological, Psychometric, and Personality Research* (2.2.5) [R Package]. Northwestern University. https://CRAN.R-project.org/package=psych

Reynolds, C. R., & Kamphaus, R. W. (2015). *Behavior Assessment System for Children* (3rd ed.). Pearson.

Roid, G. H., Miller, L. J., Pomplun, M., & Koch, C. (2013). *Leiter International Performance Scale* (3rd ed.). Stoelting Company.

Rosenthal, M. Z., Anand, D., Cassiello-Robbins, C., Williams, Z. J., Guetta, R. E., Trumbull, J., & Kelley, L. D. (2021). Development and initial validation of the Duke Misophonia Questionnaire. *Frontiers in Psychology*, *12*, Article 4197. https://doi.org/10.3389/fpsyg.2021.709928

Samajima, F. (1994). Estimation of reliability coefficients using the test information function and its modifications. *Applied Psychological Measurement*, *18*(3), 229–244. https://doi.org/10.1177/014662169401800304

Samejima, F. (1969). Estimation of latent ability using a response pattern of graded scores. *Psychometrika*, *34*(1), 1–97. https://doi.org/10.1007/bf03372160

Sapey-Triomphe, L.-A., Moulin, A., Sonié, S., & Schmitz, C. (2018). The Glasgow Sensory Questionnaire: Validation of a French language version and refinement of sensory profiles of people with high autism-spectrum quotient. *Journal of Autism and Developmental Disorders*, *48*(5), 1549–1565. https://doi.org/10.1007/s10803-017-3422-8

Schulz, S. E., & Stevenson, R. A. (2019). Sensory hypersensitivity predicts repetitive behaviours in autistic and typically-developing children. *Autism*, *23*(4), 1028–1041. https://doi.org/10.1177/1362361318774559

Simpson, K., Adams, D., Alston-Knox, C., Heussler, H. S., & Keen, D. (2019). Exploring the sensory profiles of children on the autism spectrum using the Short Sensory Profile-2 (SSP-2). *Journal of Autism and Developmental Disorders*, *49*(5), 2069–2079. https://doi.org/10.1007/s10803-019-03889-2

Sparrow, S. S. (2011). Vineland Adaptive Behavior Scales. In J. S. Kreutzer, J. DeLuca, & B. Caplan (Eds.), *Encyclopedia of clinical neuropsychology* (pp. 2618–2621). Springer. https://doi.org/10.1007/978-0-387-79948-3_1602

Sparrow, S. S., Balla, D. A., & Cicchetti, D. V. (2005). *Vineland Adaptive Behavior Scales* (2nd ed.). Pearson.

Sparrow, S. S., Cicchetti, D. V., & Saulnier, C. A. (2016). *Vineland Adaptive Behavior Scales* (3rd ed.). Pearson.

Sturm, A., Kuhfeld, M., Kasari, C., & McCracken, J. T. (2017). Development and validation of an item response theory-based Social Responsiveness Scale short form. *Journal of Child Psychology and Psychiatry*, *58*(9), 1053–1061. https://doi.org/10.1111/jcpp.12731

Tillmann, J., Uljarevic, M., Crawley, D., Dumas, G., Loth, E., Murphy, D., Buitelaar, J., Charman, T., Ahmad, J., Ambrosino, S., Auyeung, B., Baumeister, S., Beckmann, C., Bourgeron, T., Bours, C., Brammer, M., Brandeis, D., Brogna, C., de Bruijn, Y., … the AIMS-2-TRIALS LEAP group. (2020). Dissecting the phenotypic heterogeneity in sensory features in autism spectrum disorder: A factor mixture modelling approach. *Molecular Autism*, *11*(1), Article 67. https://doi.org/10.1186/s13229-020-00367-w

Toland, M. D., Sulis, I., Giambona, F., Porcu, M., & Campbell, J. M. (2017). Introduction to bifactor polytomous item response theory analysis. *Journal of School Psychology, 60*, 41–63. https://doi.org/10.1016/j.jsp.2016.11.001

Tomchek, S. D., Huebner, R. A., & Dunn, W. (2014). Patterns of sensory processing in children with an autism spectrum disorder. *Research in Autism Spectrum Disorders*, *8*(9), 1214–1224. https://doi.org/10.1016/j.rasd.2014.06.006

Turner-Brown, L. M., Lam, K. S. L., Holtzclaw, T. N., Dichter, G. S., & Bodfish, J. W. (2011). Phenomenology and measurement of circumscribed interests in autism spectrum disorders. *Autism*, *15*(4), 437–456. https://doi.org/10.1177/1362361310386507

Uljarević, M., Frazier, T. W., Jo, B., Scahill, L., Youngstrom, E. A., Spackman, E., Phillips, J. M., Billingham, W., & Hardan, A. (2023). Dimensional Assessment of Restricted and Repetitive Behaviors: Development and preliminary validation of a new measure. *Journal of the American Academy of Child & Adolescent Psychiatry*, *62*(5), 568–581. https://doi.org/10.1016/j.jaac.2022.07.863

Uljarević, M., Jo, B., Frazier, T. W., Scahill, L., Youngstrom, E. A., & Hardan, A. Y. (2021). Using the big data approach to clarify the structure of restricted and repetitive behaviors across the most commonly used autism spectrum disorder measures. *Molecular Autism*, *12*(1), Article 39. https://doi.org/10.1186/s13229-021-00419-9

Watson, L. R., Patten, E., Baranek, G. T., Poe, M., Boyd, B. A., Freuler, A., & Lorenzi, J. (2011). Differential associations between sensory response patterns and language, social, and communication measures in children with autism or other developmental disabilities. *Journal of Speech, Language, and Hearing Research*, *54*(6), 1562–1576. https://doi.org/10.1044/1092-4388(2011/10-0029)

Wechsler, D. (2011). *Wechsler Abbreviated Scale of Intelligence: WASI-II* (2nd ed.). Pearson.

Wilcox, R. R., & Rousselet, G. A. (2018). A guide to robust statistical methods in neuroscience. *Current Protocols in Neuroscience*, *82*, 8.42.1-8.42.30. https://doi.org/10.1002/cpns.41

Williams, Z. J. (2020). Short Sensory Profile in autism. In F. R. Volkmar (Ed.), *Encyclopedia of autism spectrum disorders* (2nd ed., pp. 1–7). Springer. https://doi.org/10.1007/978-1-4614-6435-8_102311-1

Williams, Z. J., Failla, M. D., Gotham, K. O., Woynaroski, T. G., & Cascio, C. (2018). Psychometric evaluation of the Short Sensory Profile in youth with autism spectrum disorder. *Journal of Autism and Developmental Disorders*, *48*(12), 4231–4249. https://doi.org/10.1007/s10803-018-3678-7
